# Supplementary material for: Spatial probabilistic mapping of metabolite ensembles in mass spectrometry imaging
Source: Nat Commun. 2023 Apr 1;14:1823. doi: 10.1038/s41467-023-37394-z (PMC10067847; doi:10.1038/s41467-023-37394-z)
Supplement: Supplementary file 1 — Supplementary Information [file 41467_2023_37394_MOESM1_ESM.pdf]

Supplementary Information

Spatial Probabilistic Mapping of Metabolite Ensembles in Mass Spectrometry Imaging

Denis Abu Sammour [1,2], James L. Cairns [1,3], Tobias Boskamp [4,5], Christian Marsching [1,4], Tobias Kessler [6,7], Carina Ramallo Guevara [1], Verena Panitz [7,8], Ahmed Sadik [7,9], Jonas Cordes [2,10], Stefan Schmidt [1], Shad A. Mohammed [1,2], Miriam F. Rittel [1,2], Mirco Friedrich [11,12], Michael Platten [11,12], Ivo Wolf [2,10], Andreas von Deimling [13], Christiane A. Opitz [7,8], Wolfgang Wick [6,7], Carsten Hopf [1,2,3*]

[1] Center for Mass Spectrometry and Optical Spectroscopy (CeMOS), Mannheim University of Applied Sciences, Mannheim, Germany

[2] Institute for Medical Technology, Heidelberg University and Mannheim University of Applied Sciences, Mannheim, Germany

[3] Medical Faculty Heidelberg, Heidelberg University, Heidelberg, Germany

[4] Bruker Daltonics GmbH & Co. KG, Bremen, Germany

[5] Center for Industrial Mathematics, University of Bremen, Bremen, Germany

[6] Clinical Cooperation Unit Neurooncology, German Cancer Consortium, German Cancer Research Center, Heidelberg, Germany

[7] DKTK Metabolic Crosstalk in Cancer, German Consortium of Translational Cancer Research (DKTK), German Cancer Research Center (DKFZ), Heidelberg, Germany

[8] Department of Neurology and National Center for Tumor Diseases, Heidelberg University Hospital, Heidelberg, Germany

[9] Faculty of Bioscience, Heidelberg University, Heidelberg, Germany

[10] Faculty of Computer Science, Mannheim University of Applied Sciences, Mannheim, Germany

[11] Department of Neurology, MCTN, Medical Faculty Mannheim, Heidelberg University, Mannheim, Germany.

[12] DKTK Clinical Cooperation Unit Neuroimmunology and Brain Tumor Immunology, DKFZ, Heidelberg, Germany.

[13] Department of Neuropathology, University Hospital Heidelberg, and, Clinical Cooperation Unit Neuropathology, German Cancer Research Center (DKFZ), German Cancer Consortium, Heidelberg, Germany

* Correspondence should be addressed to C.H. (c.hopf@hs-mannheim.de).

Table of Contents

Supplementary Figures 4
Supplementary Fig. 1. 4
Supplementary Fig. 2. 5
Supplementary Fig. 3. 6
Supplementary Fig. 4. 7
Supplementary Fig. 5. 8
Supplementary Fig. 6. 9
Supplementary Fig. 7. 10
Supplementary Fig. 8. 11
Supplementary Fig. 9. 12
Supplementary Fig. 10. 13
Supplementary Fig. 11. 14
Supplementary Fig. 12. 15
Supplementary Fig. 13. 16
Supplementary Fig. 14. 17
Supplementary Fig. 15. 18
Supplementary Fig. 16. 19
Supplementary Fig. 17. 20
Supplementary Fig. 18. 21
Supplementary Fig. 19. 22
Supplementary Fig. 20. 23
Supplementary Fig. 21. 24
Supplementary Fig. 22. 25
Supplementary Fig. 23. 26
Supplementary Fig. 24. 27
Supplementary Fig. 25. 28
Supplementary Fig. 26. 29
Supplementary Fig. 27. 30
Supplementary Fig. 28. 31
Supplementary Fig. 29. 32
Supplementary Fig. 30. 33

Supplementary Fig. 31. 34
Supplementary Fig. 32. 35
Supplementary Fig. 33. 36
Supplementary Fig. 34. 37
Supplementary Tables 39
Supplementary Table 1. 39
Supplementary References 40

Supplementary Figures

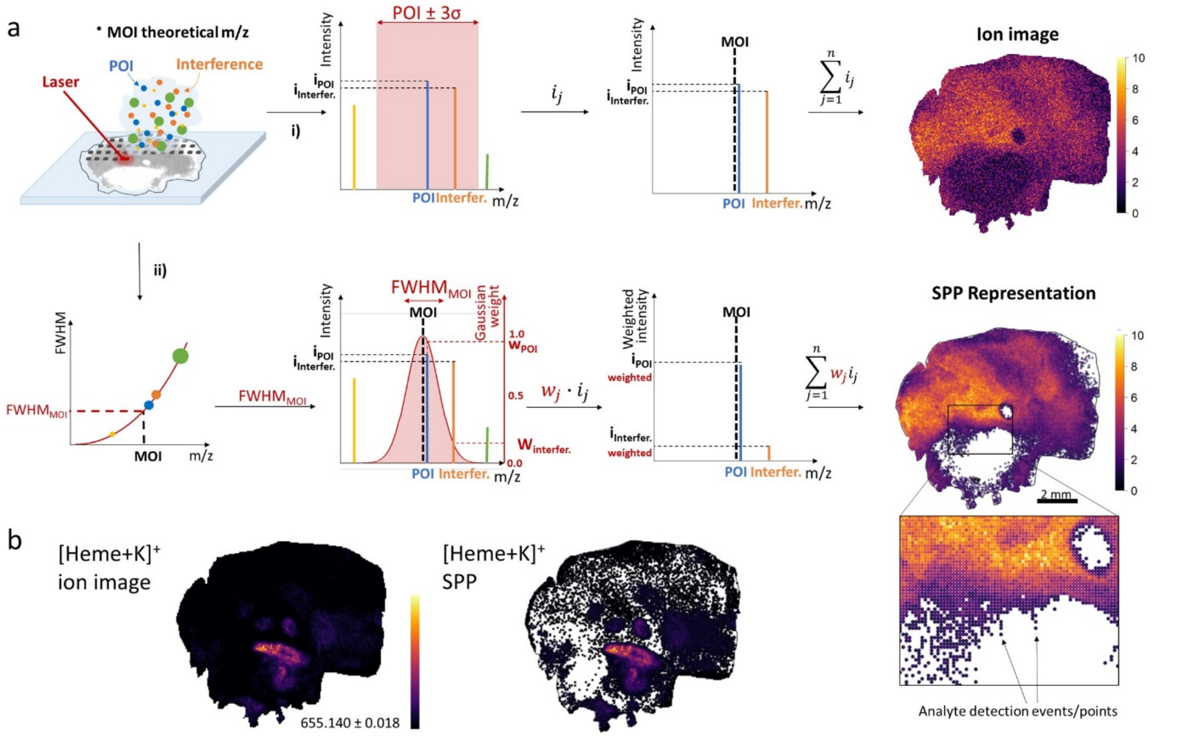

Supplementary Fig. 1. Comparison of uniform intensity-weighted ion image versus FWHM-dependent Gaussian-weighted **spatial point pattern (SPPs)** concepts. Both are representations of an observed **Peak-of-Interest** (**POI**; with observed *m/z*), additional peaks ("interference") within a narrow mass range around that POI, and a database-annotated **Metabolite-of-interest** (**MOI**; black dashed line; with known *m/z*), that users may be interested in based on their research agenda. POI and MOI *m/z* generally differ somewhat. SPPs are the mathematical basis of moleculaR that enable the subsequent probabilistic spatial mapping of the MOI (**Fig. 2a**). **a)** i) Ion images are typically generated by summing up (with identical weights) all observed ion intensities (blue and orange vertical lines) within a (typically user-defined) mass-range (pink area) around the POI *m/z* that covers also the MOI. ii) SPP representation of the same MOI. A single full profile mass spectrum is used to compute full width at half-maximum (FWHM) values of all its peaks across the *m/z* axis. A curve is fitted to describe the continuous relation of FWHM as a function of *m/z*. A Gaussian envelope, whose sigma is inferred from the FWHM model at MOI *m/z*, is centered at that *m/z* MOI. The Gaussian is then used as a weighting factor to protect against proximal background signals, i.e. interferences; the further the theoretical *m/z* the user is interested in (= MOI) is from the measured *m/z* (= POI), the lower the weight that POI receives in the SPP representation. The resulting SPP map represents weighted points/events distributed in a 2D window, i.e. the tissue section. **b)** An exemplary comparison between conventional ion image and SPP representation of [Heme + K]$^+$. White points in SPPs indicate absence of the MOI signal.

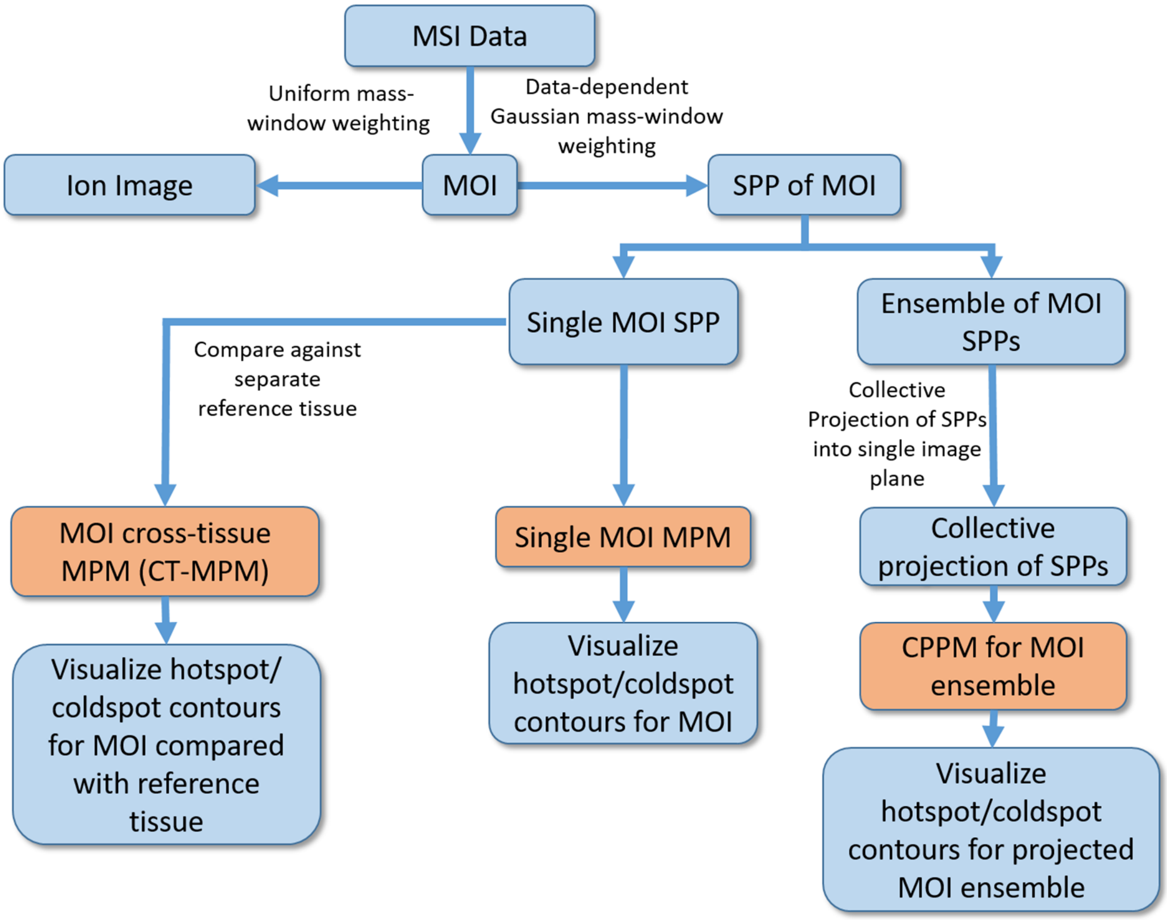

Supplementary Fig. 2. Top-level overview of different analyses introduced by *moleculaR*. Based on a Gaussian-weighted spatial point pattern (SPP) representation of a single MOI, a **molecular probabilistic map (MPM)** is computed for that MOI. This probabilistic map visualizes areas of statistically different relative abundance or deficiency of that MOI, which are visualized as "hotspot" and "coldspot" contours, respectively. Entire sets of SPPs representing an ensemble of MOIs such as a class of phospholipids can be collectively projected into a single image plane. A **collective projection probabilistic map** (**CPPM**) is then computed for the collective projection SPP (CP-SPP) representing this ensemble of MOI. Finally, MPMs for single MOI can be cross-referenced against a reference tissue (e.g., a tumor test sample against a non-tumor reference sample) to generate **cross-tissue molecular probabilistic maps** (**CT-MPM**).

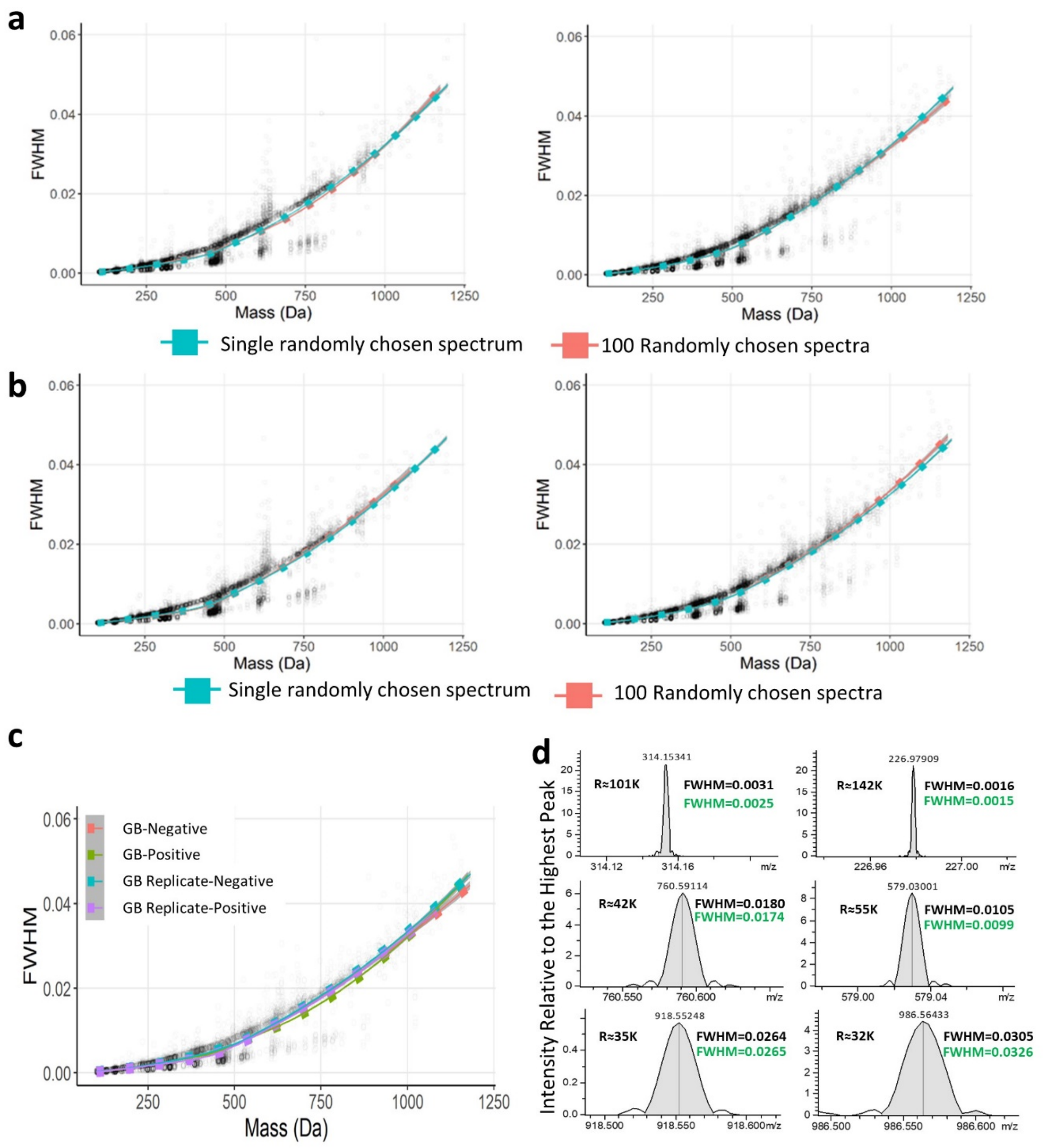

Supplementary Fig. 3. FWHM as a function of mass was calculated for MALDI-FTICR-MSI data for the human glioblastoma (GB) tissue section of **Fig. 3ab** and its serial section as a technical replicate (**Supplementary Fig. 34**). **a)** FWHM curves were fit via locally estimated scatterplot smoothing (loess) for this GB tissue data based on a single versus 100 randomly chosen spectra for the positive ion- (left panel) and negative ion (right panel) modes. **b)** FWHM curves for the GB tissue replicate data. **c)** Superimposition of single randomly chosen spectra (positive ion and negative ion modes) for each of the FWHM curves shown in **a** and **b**. **d)** Six arbitrary peaks taken from the positive ion (left column) and negative ion (right column) mode spectra in **c**. The corresponding FWHM values (black) are displayed as provided by the vendor software (DataAnalysis version 5.3; Bruker Daltonics) and as estimated based on the fitted FWHM curves (green). Source data are provided as a Source Data file.

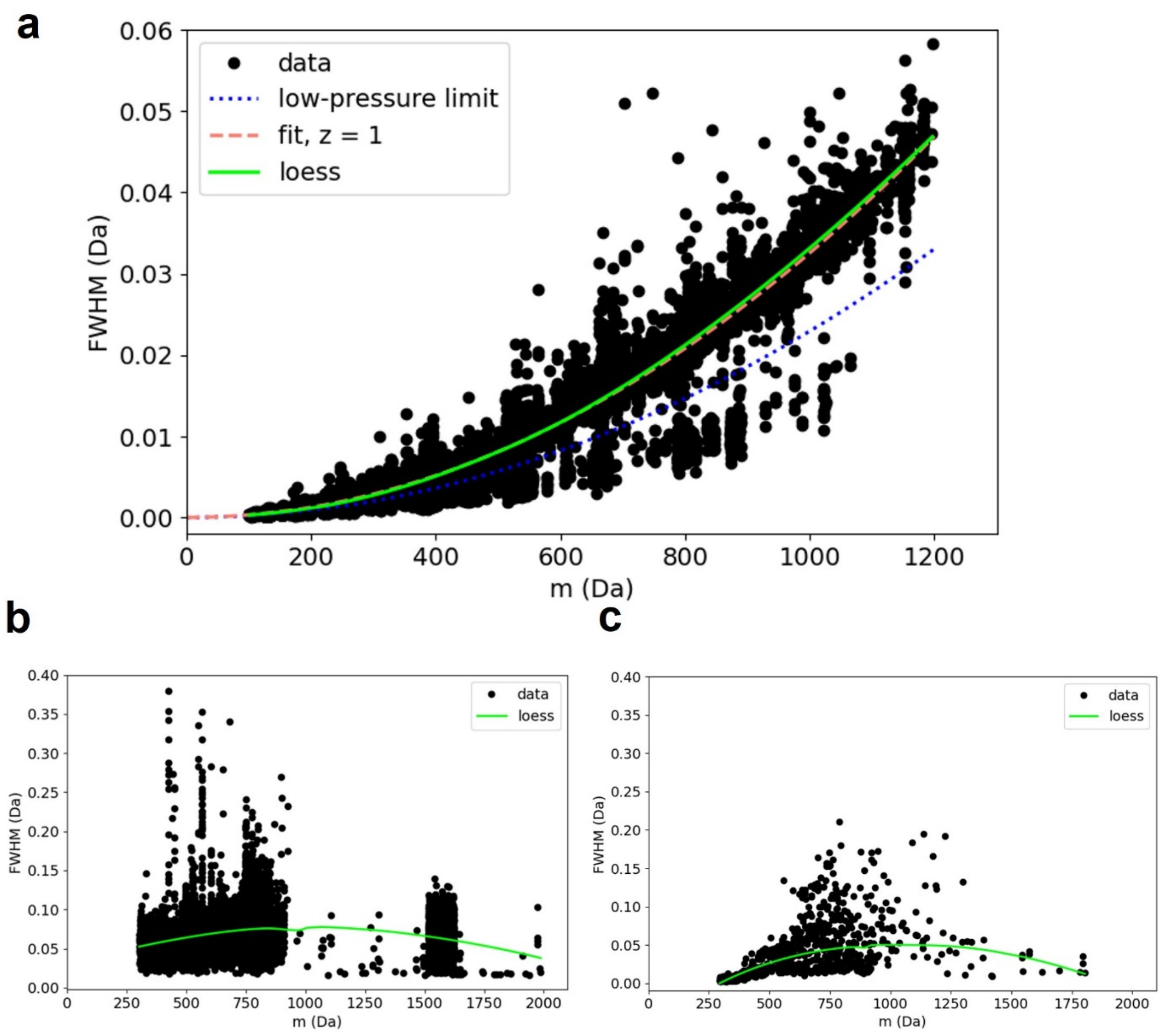

Supplementary Fig. 4. Comparison of FWHM as a function of ion mass curves for different MSI modalities estimated from MALDI MSI data acquired on **a)** FTICR (magnetic resonance) MS, **b)** rapifleX TOF and **c)** timsTOFflex mass spectrometers. For every case, 100 randomly chosen mass spectra were evaluated for the FWHM curve via locally estimated scatterplot smoothing (loess; green curve). **a)** Comparison between the calculated theoretical FWHM vs mass relationship excluding ion collisions (low-pressure limit; dashed blue curve; equation 2 in **Methods**) and including ion collisions (dashed pink curve; equation 3 in **Methods**; z is the ion charge) on the one hand, and the empirical FWHM vs *m/z* based on the estimated FWHM values (solid green curve; loess) for the MALDI FTICR MSI data on the other hand. The estimated FWHM model based on empirical data closely follows the FWHM theoretical expectation for the mass spectrometer. Source data are provided as a Source Data file.

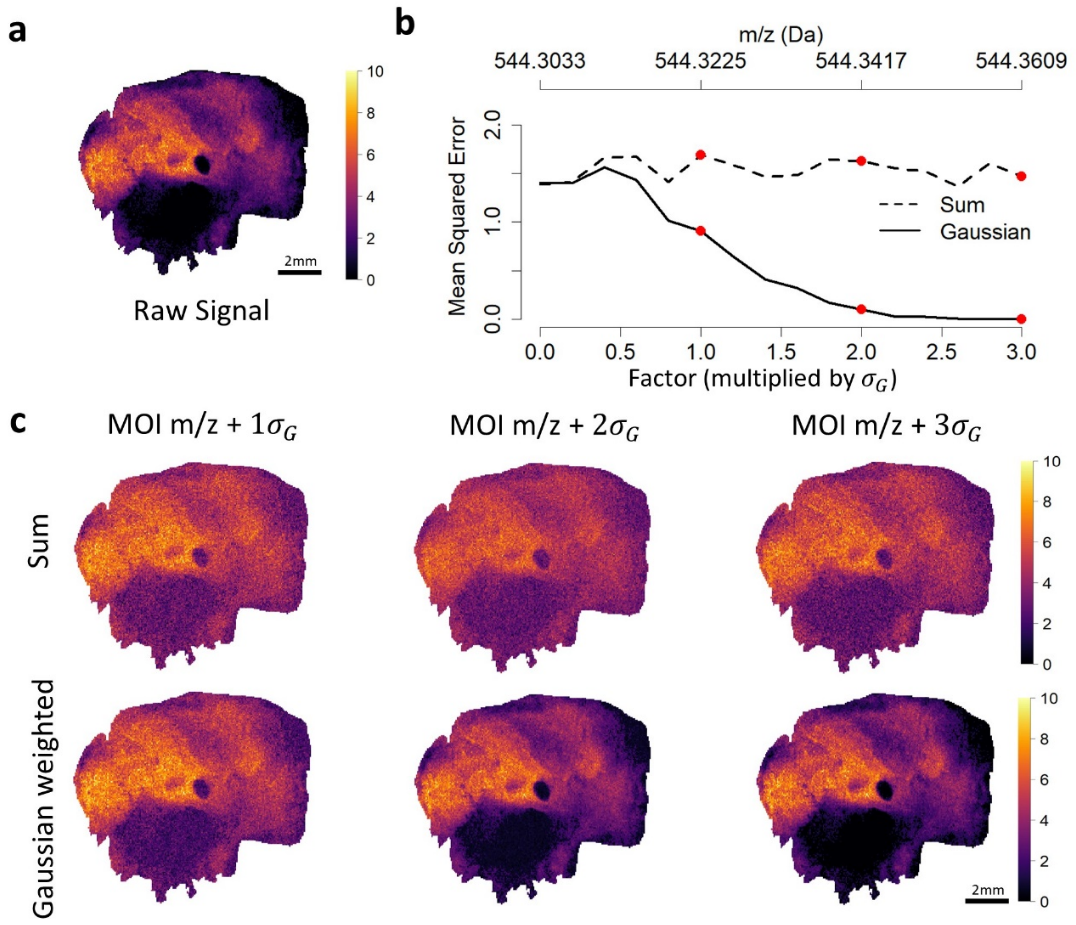

Supplementary Fig. 5. The effect of the Gaussian mass-window weighting method of **Fig. 1** on an exemplary MOI, PE(20:1)[M+Na]$^+$ (monoisotopic *m/z* 544.3009; FDR≤0.2), „contaminated" with an artificial noise source as in **Fig. 4b** (interference type, i.e. spiked at a close proximity of *m/z* MOI). **a)** Raw ion image of *m/z* 544.3009 of the GB tissue section in **Fig. 2a** (estimated FWHM = 0.0082; corresponding σ_G= 0.0193). **b)** Pixel-wise mean squared error (MSE) between raw data image in **a** and artificially contaminated data image computed for increasing *m/z* distances between raw and contaminated data measured in multiples of σ_G away from MOI m/z (MOI + Factor(σ_G)). Compared to the standard uniform mass-window weighting (indicated as "sum"; dashed line) the Gaussian weighting method handles interference noise (modelling proximal background signals) more effectively, which is also optically evident in the lower panel of **c**. Source data are provided as a Source Data file.

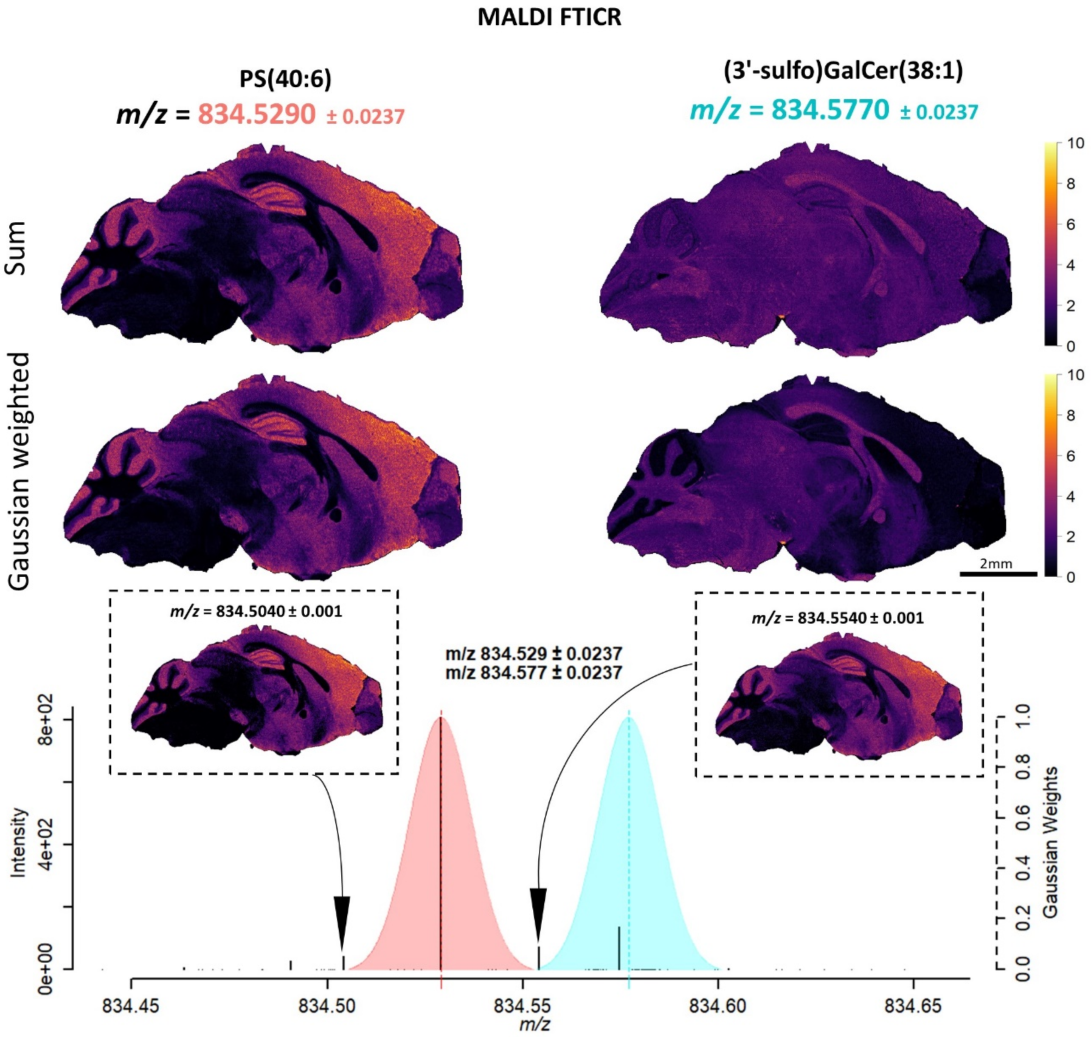

Supplementary Fig. 6. Comparison of Gaussian mass-window weighting effects on FTICR-, timsTOF- and TOF MALDI MSI data (**Supplementary Fig. 6-Supplementary Fig. 8**) – here FTICR MSI. Gaussian mass-window weighting (FWHM = 0.0186; σ_G = 0.0079; $3\sigma_G$ = 0.0237; mass resolving power at *m/z* 834.5290 ≅ 45,000) of exemplary MOIs, phosphatidylserine PS(40:6) $[M\text{-}H]^-$ (MOI1; *m/z* 834.5290; FDR ≤0.05; light red) and C20:0 sulfatide (3'-sulfo) GalCer(38:1)$[M\text{-}H]^-$ (MOI2; *m/z* 834.5770; FDR ≤0.10; turquoise) shown for a mouse brain MALDI-FTICR-MSI data. The mass spectrum in the lower panel shows a centroided overview sum spectrum generated by summing up all peak intensities throughout the entire dataset. The color-coded Gaussians represent the Gaussian mass-window weighting envelopes for the above mentioned MOIs (red dotted line for MOI1 and turquoise dotted line for MOI2). The mass window width is inferred from the mass resolving power at the respective MOI and taken as the calculated *m/z* MOI ± $3\sigma_G$ (i.e. the span of each Gaussian envelope width of the colored areas of each MOI individually) for both uniform mass-window weighting ("sum"; upper row) and the Gaussian mass-window weighting (middle row). Please note the two side lobe peaks [1] (black arrows) of MOI1 with the one at *m/z* 834.5540 falling within the calculated mass window of MOI2. This interfering peak results in a homogenous intensity distribution for MOI2 throughout the tissue section based on the uniform window weighting ("sum"), while the Gaussian–weighted method successfully down-weights this interference, as indicated by the distinguishable intensity images for both MOIs.

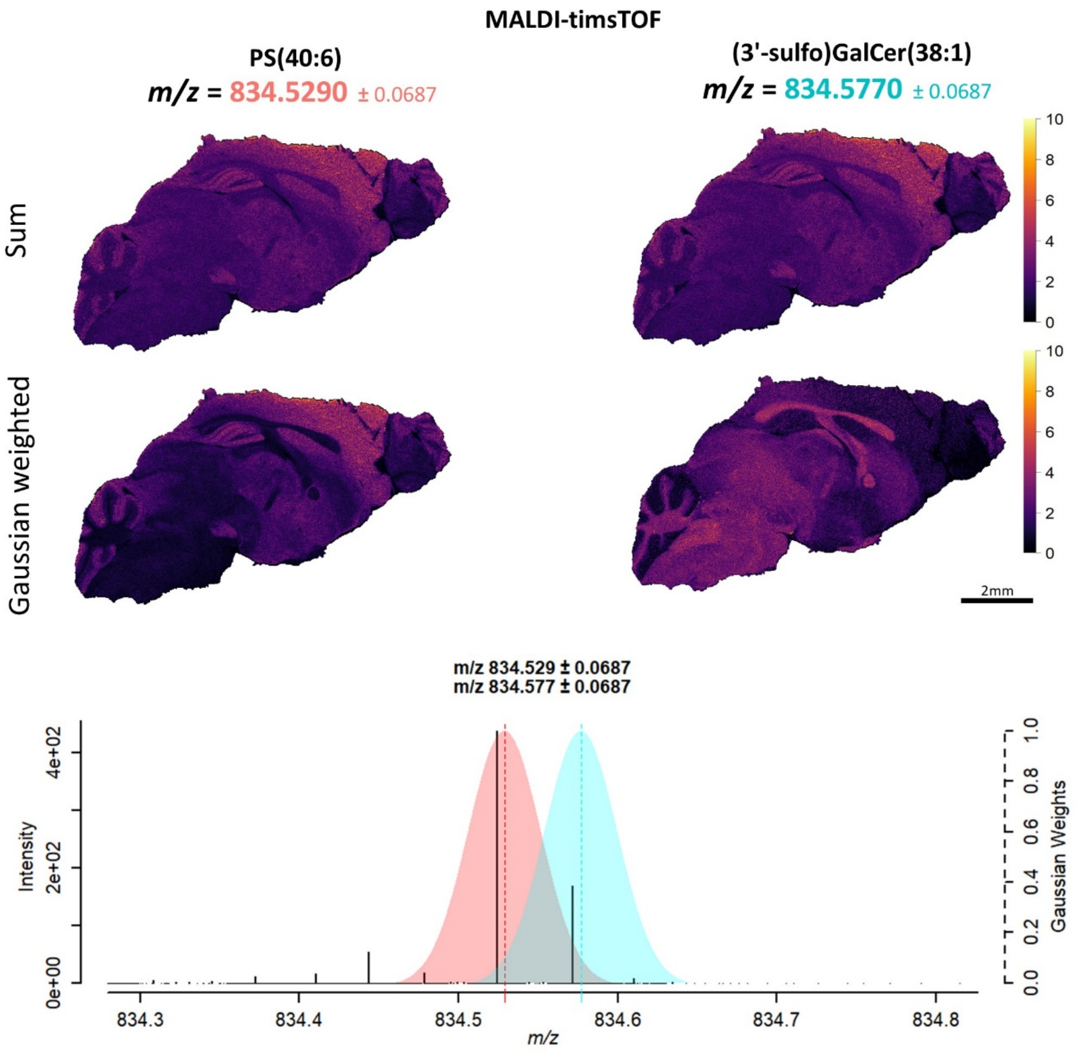

Supplementary Fig. 7. Comparison of Gaussian mass-window weighting effects on FTICR-, timsTOF- and TOF data – here timsTOF MALDI-MSI. Gaussian mass-window weighting (FWHM = 0.0539; σ_G = 0.0229; $3\sigma_G$ = 0.0687; mass resolving power at *m/z* 834.5290 ≅ 15,000) of exemplary MOIs, phosphatidylserine PS(40:6)[M-H]$^-$ (MOI1; *m/z* 834.5290; FDR ≤0.05; light red) and C20:0 sulfatide (3'-sulfo) GalCer(38:1)[M-H]$^-$ (MOI2; *m/z* 834.5770; FDR ≤0.10; turquoise) shown for a mouse brain MALDI-timsTOF-MSI data. The mass spectrum in the lower panel shows a centroided overview sum spectrum generated by summing up all peak intensities throughout the entire dataset. The color-coded Gaussians represent the Gaussian mass-window weighting envelopes for the above-mentioned MOIs (red dotted line for MOI1 and turquoise dotted line for MOI2). The mass window width is inferred from the mass resolving power at the respective MOI and taken as the calculated *m/z* MOI ± $3\sigma_G$ (i.e. the span of each Gaussian envelope width of the colored areas of each MOI individually) for both uniform mass-window weighting ("sum"; upper row) and the Gaussian mass-window weighting (middle row). The results indicate that Gaussian-weighting is able to downplay the effects of proximal interfering peaks.

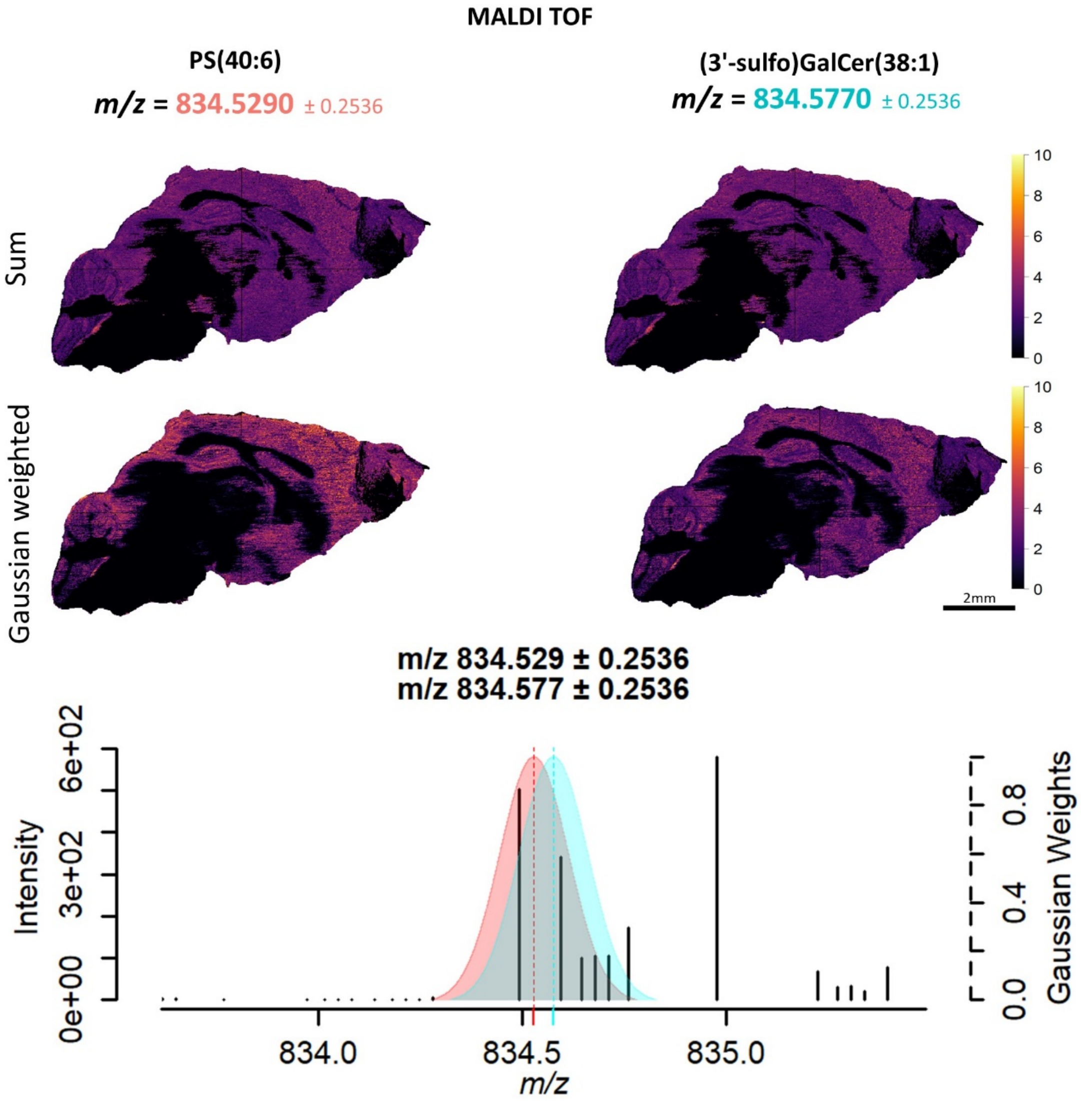

Supplementary Fig. 8. Comparison of Gaussian mass-window weighting effects on FTICR-, timsTOF- and TOF data – here TOF MSI. Gaussian mass-window weighting (FWHM = 0.1991; σ_G = 0.0845; $3\sigma_G$ = 0.2536; mass resolving power at *m/z* 834.5290 ≅ 5,000) of exemplary MOIs, phosphatidylserine PS(40:6)[M-H]$^-$ (MOI1; *m/z* 834.5290; FDR ≤0.05; light red) and C20:0 sulfatide (3'-sulfo) GalCer(38:1) [M-H]$^-$ (MOI2; *m/z* 834.5770; FDR ≤0.10; turquoise) shown for sub-standard (used on purpose) mouse brain MALDI-TOF-MSI data. The mass spectrum in the lower panel shows a centroided overview sum spectrum generated by summing up all peak intensities throughout the entire dataset. The color-coded Gaussians represent the Gaussian mass-window weighting envelopes for the above mentioned MOIs (red dotted line for MOI1 and turquoise dotted line for MOI2). The mass window width is inferred from the mass resolving power at the respective MOI and taken as the calculated *m/z* MOI ± $3\sigma_G$ (i.e. the span of each Gaussian envelope width of the colored areas of each MOI individually) for both uniform mass-window weighting ("sum"; upper row) and the Gaussian mass-window weighting (middle row). The mass resolution of this measurement was insufficient for distinguishing the mentioned MOIs as was possible with MALDI-FTICR-MSI (**Supplementary Fig. 6**) and MALDI-timsTOF-MSI (**Supplementary Fig. 7**).

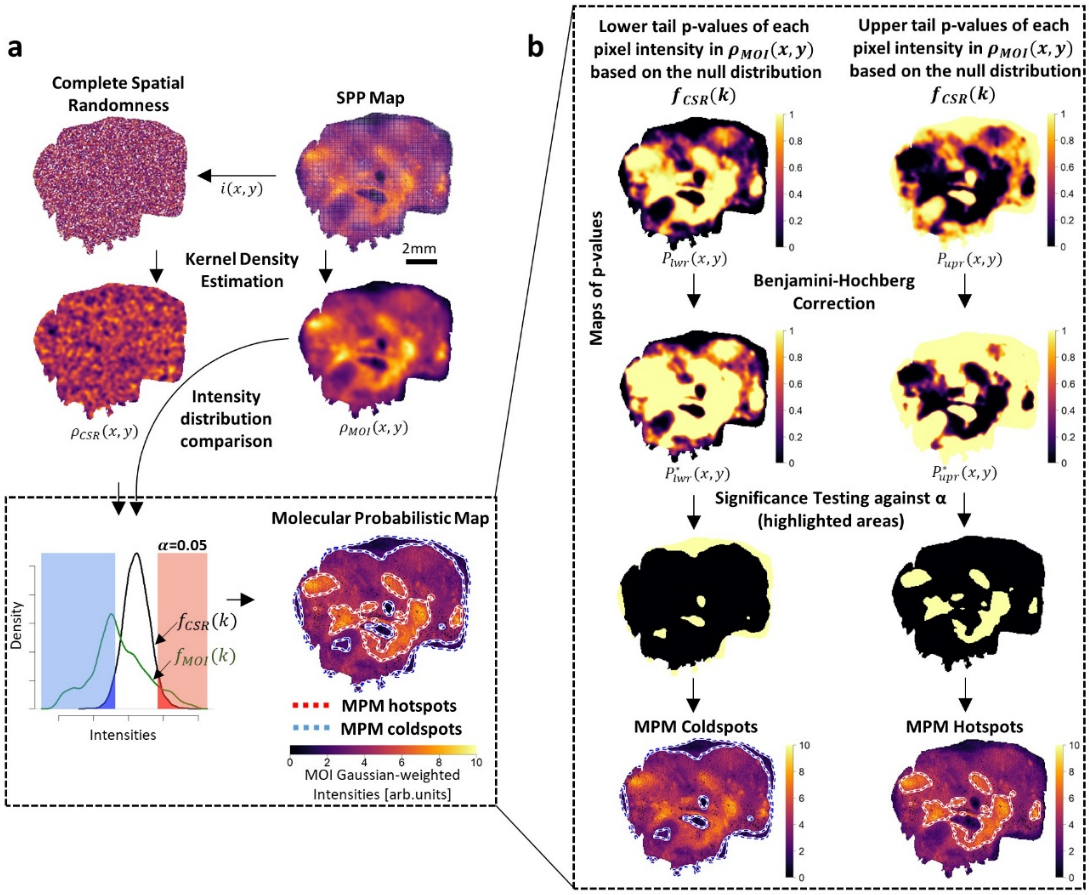

Supplementary Fig. 9. Computational Molecular Probabilistic Map (MPM) workflow. **a)** A corresponding complete spatial randomness (CSR) model is created for each metabolite-of-interest's (MOI; here SM(d36:4)[M+H$^+$]; FDR ≤0.10; FTICR MSI) spatial point pattern (SPP) with equal spatial point density. Kernel density is estimated for both, thus resulting in spatial density functions, $\rho_{\mathrm{MOI}}(x,y)$ and $\rho_{\mathrm{CSR}}(x,y)$. The intensity distribution function $f_{\mathrm{CSR}}(k)$, which converges to a normal distribution (**Supplementary Fig. 12**), then serves as the null distribution against which the pixel intensities of $\rho_{\mathrm{MOI}}(x,y)$ are tested. **b)** For each pixel intensity value in $\rho_{\mathrm{MOI}}(x,y)$ a lower and upper tail p-value is computed based on the null distribution $f_{\mathrm{CSR}}(k)$ resulting in two spatial maps of lower and upper tail p-values $P_{\mathrm{lwr}}(x,y)$ and $P_{\mathrm{upr}}(x,y)$, respectively. These p-values are then Benjamini-Hochberg corrected and null-hypothesis significance testing is carried out against a significance level α of 0.05. MPM hotspots (red/white contours) and coldspots (blue/white contours) are accordingly defined as locations where the null-hypothesis is rejected for the upper or lower tail corrected p-values, thus signifying areas of significant MOI relative spatial abundance and deficiency, respectively.

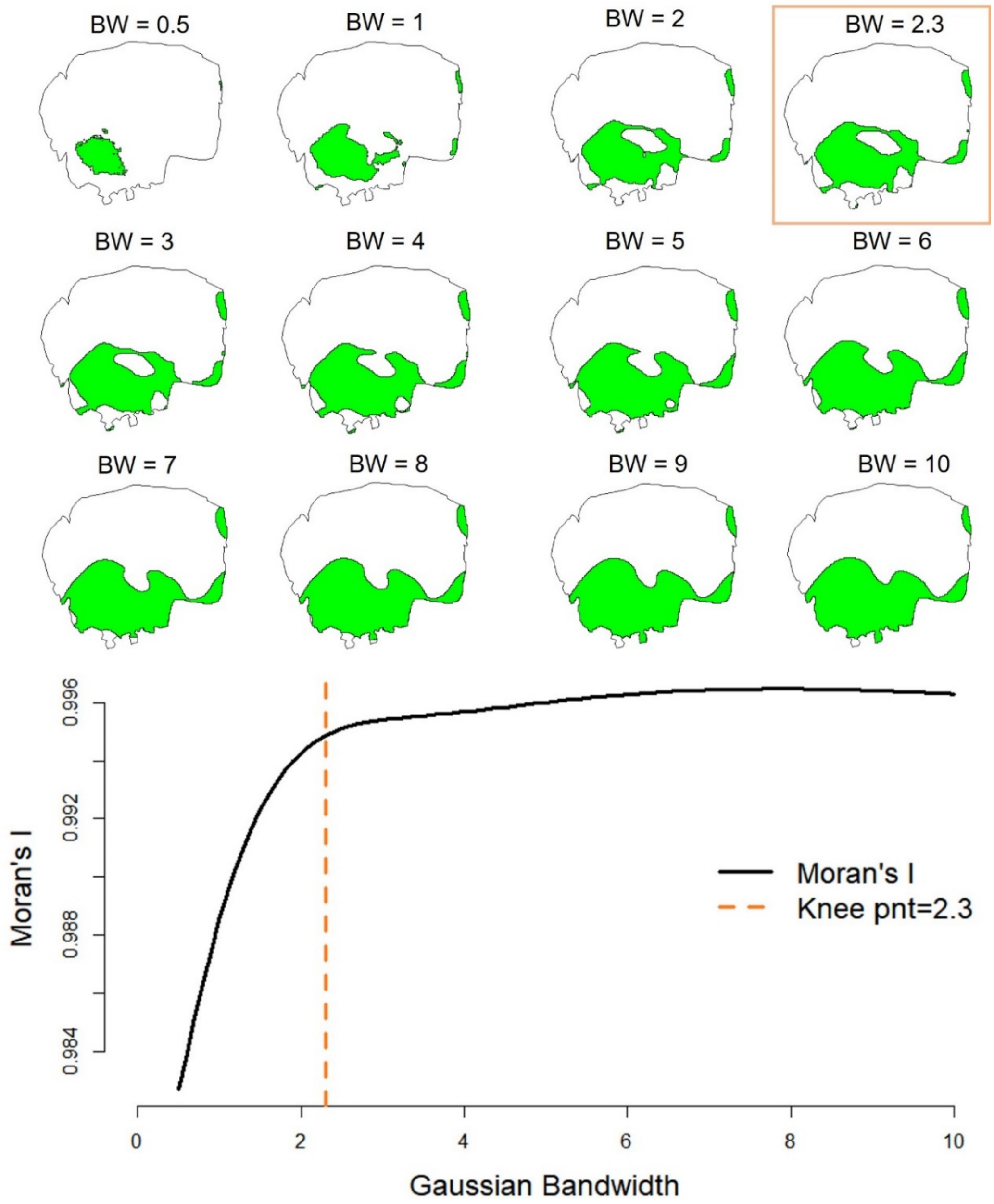

Supplementary Fig. 10. Bandwidth estimation for the kernel density estimation (KDE) procedure as part of the MPM workflow (**Fig. 1a**) evaluated for putative PS(36:1)[M-H]$^-$ at *m/z* 788.5447 (**Fig. 3b**). The KDE bandwidth h_{KDE} is varied iteratively from values of 1 to 10 (i.e., pixels; multiples of 50 µm) in 0.5 incremental steps. During each iteration KDE is applied, and the Moran's I statistic, a measure of autocorrelation, is determined. The optimal h_{KDE} is then determined by finding the point in the Moran's I vs bandwidth $\mathrm{h_{KDE}}$ plot, at which the spatial autocorrelation levels-off, i.e. after which an increase in h_{KDE} does not result in a considerable increase in the spatial autocorrelation of the smoothed density image. This "knee" point is estimated via the kneedle method [2]. Source data are provided as a Source Data file.

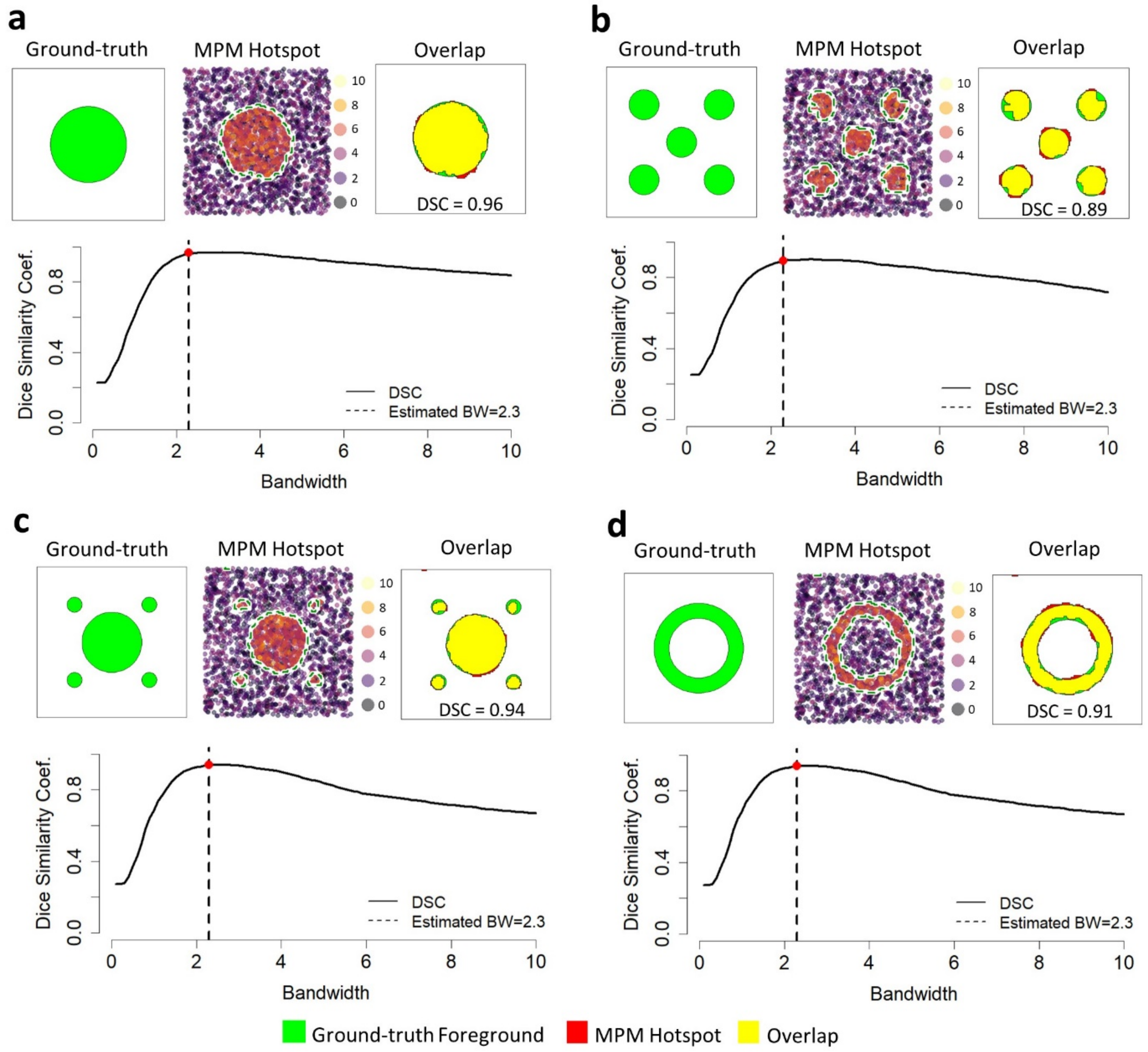

Supplementary Fig. 11. Use of simulated data (**Fig. 2b** and **Supplementary Fig. 13)** for evaluating the proposed KDE bandwidth estimation method (**Supplementary Fig. 10; Methods**) as part of the MPM workflow (**Fig. 1a**). **a to d,** for each of the four different ground-truth shapes, an SPP was simulated as described in **Fig. 2b** and **Supplementary Fig. 13**. The corresponding MPM was estimated with the KDE bandwidth estimation method shown in **Supplementary Fig. 10**. Finally, the Dice similarity coefficient (DSC) was computed between the estimated MPM hotspot contour (red) and the ground-truth foreground (green; indicating high abundance), based on which the simulated SPP was created. Estimated DSCs of all simulated ground-truths showed high degree of overlap (yellow) at 0.96, 0.89, 0.94 and 0.91 for **a**, **b**, **c** and **d**, respectively. Moreover, MPM was iteratively estimated for every simulated SPP type using assigned KDE Gaussian bandwidth h_{KDE} , which was varied from values of 0 to 10 length units in 0.1 incremental steps. During each iteration, DSC was computed between the MPM hotspot contour and the ground-truth foreground used for generating the simulated SPP. The resulting curves suggest that the KDE bandwidth estimation method of MPM coincides with the highest (i.e. optimum) DSC for all simulated SPPs. Source data are provided as a Source Data file.

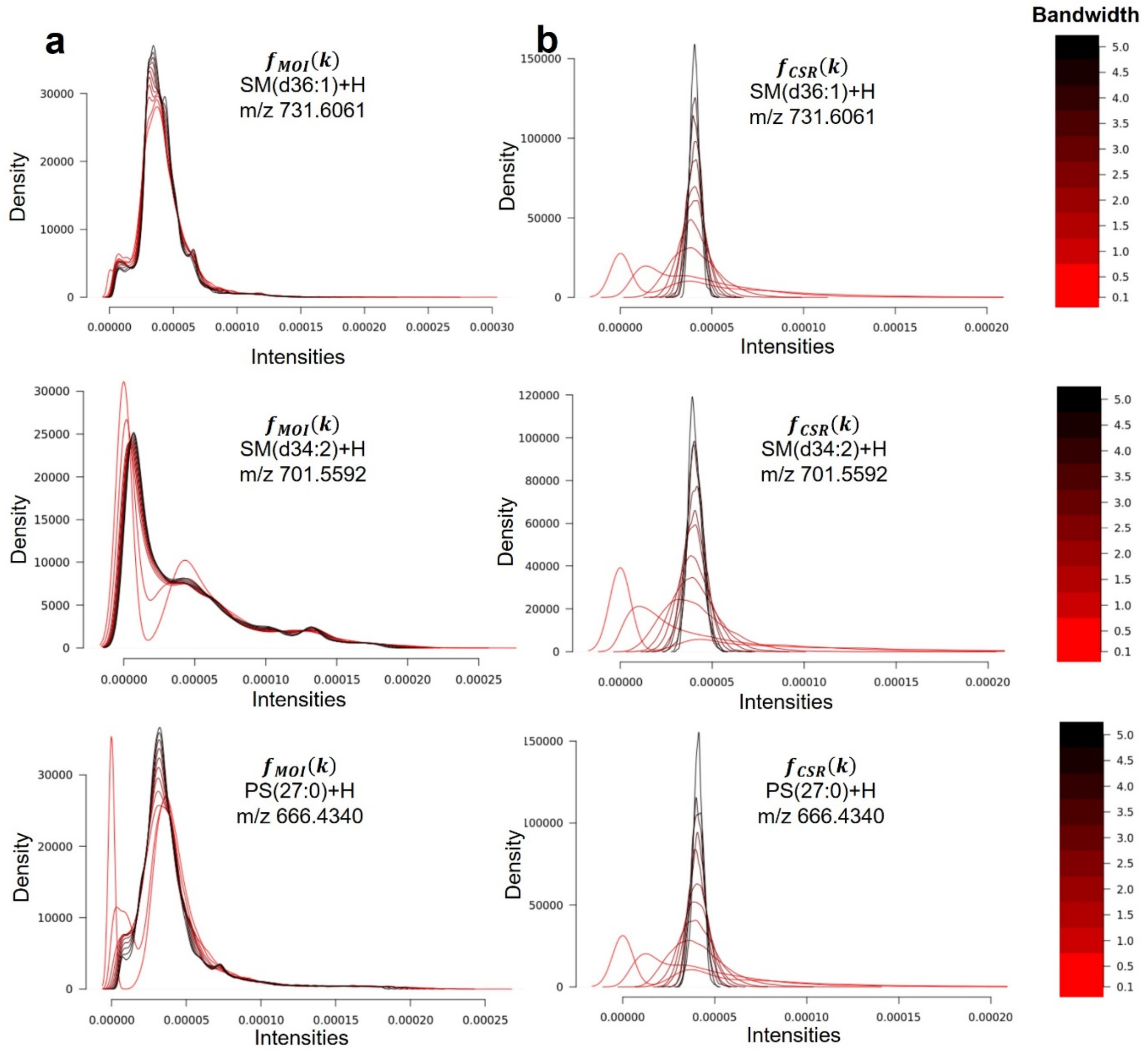

Supplementary Fig. 12. Intensity distributions $f_{\mathrm{MOI}}(k)$ (column **a**) and $f_{\mathrm{CSR}}(k)$ (column **b**) corresponding to the 2D spatial densities of the MOI, $\rho_{\mathrm{MOI}}(x,y)$, and of the complete spatial randomness (CSR) model, $\rho_{\mathrm{CSR}}(x,y)$, respectively, of three different *m/z* that putatively correspond to the indicated lipids (rows). While $f_{\mathrm{MOI}}(k)$ does not necessarily converge onto a normal distribution for the range of bandwidths under consideration (**a**), as a consequence of the central limit theorem, the corresponding intensity distribution $f_{\mathrm{CSR}}(k)$ approximates a normal distribution as the bandwidth increases, even for smaller bandwidth values (**b**).

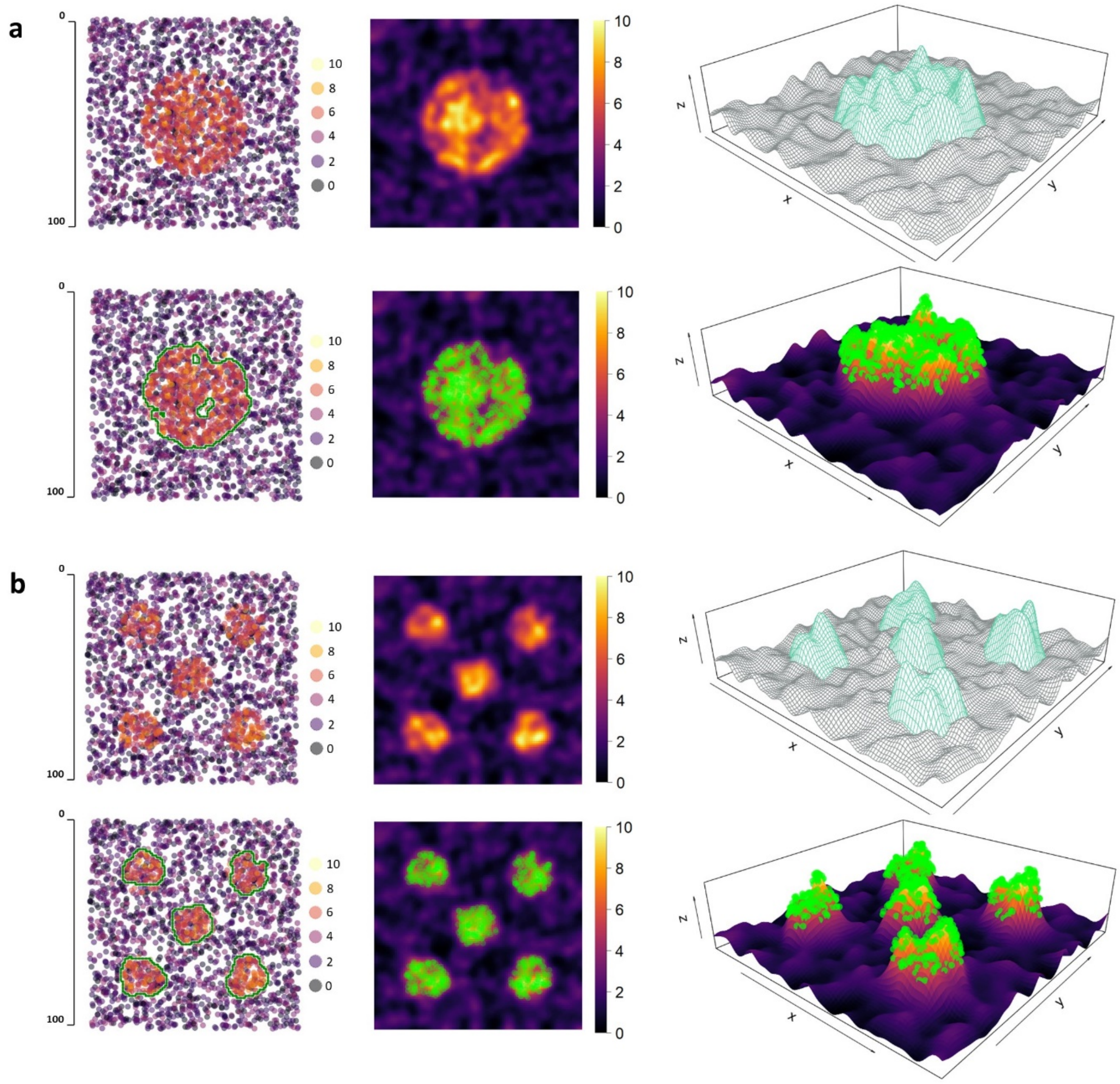

Supplementary Fig. 13. Molecular probabilistic maps (MPMs) correctly assign significant relative spatial abundance/ deficiency in simulated data. **a)** Synthetic uniform Poisson SPP simulating an MOI spatial ground-truth with a circular central circle of radius 25 length units representing high MOI abundance embedded in a background (upper panel; left; see also **Supplementary Fig. 11a**). Intensity values were sampled from above and below the upper quartile of the empirical intensities of a MALDI-FTICR-MSI measurement of a human GB tissue sample (**Fig. 3a**) at *m/z* 544.3009 (PE(20:1)$[M+Na]^+$; FDR ≤ 0.2) for the high-MOI area and background, respectively (high-MOI area *n*≈570 with spatial point density of ~0.4 points per unit area; background *n*≈1970 with spatial point density of ~0.3 points per unit area; mean signal intensity of high-MOI area/mean signal intensity of background ≈2.3). The corresponding spatial density image (upper panel; middle) and 3D surface plots (upper panel; right) are shown. MPM computation was able to localize the simulated high-MOI area correctly (green contours; lower panel; left) and identify points exhibiting significant relative spatial abundance (green points; lower panel; middle and right). **b)** Simulated SPP for an MOI spatial ground-truth similar to **a** with five identical circular high-MOI areas of 10 length units radius (simulated high-MOI area *n*≈470; background *n*≈1950; same spatial point density as in **b**; mean signal intensity of high-MOI area/mean signal intensity of background ≈2.3). MPM computation was able to localize all simulated high-MOI areas correctly.

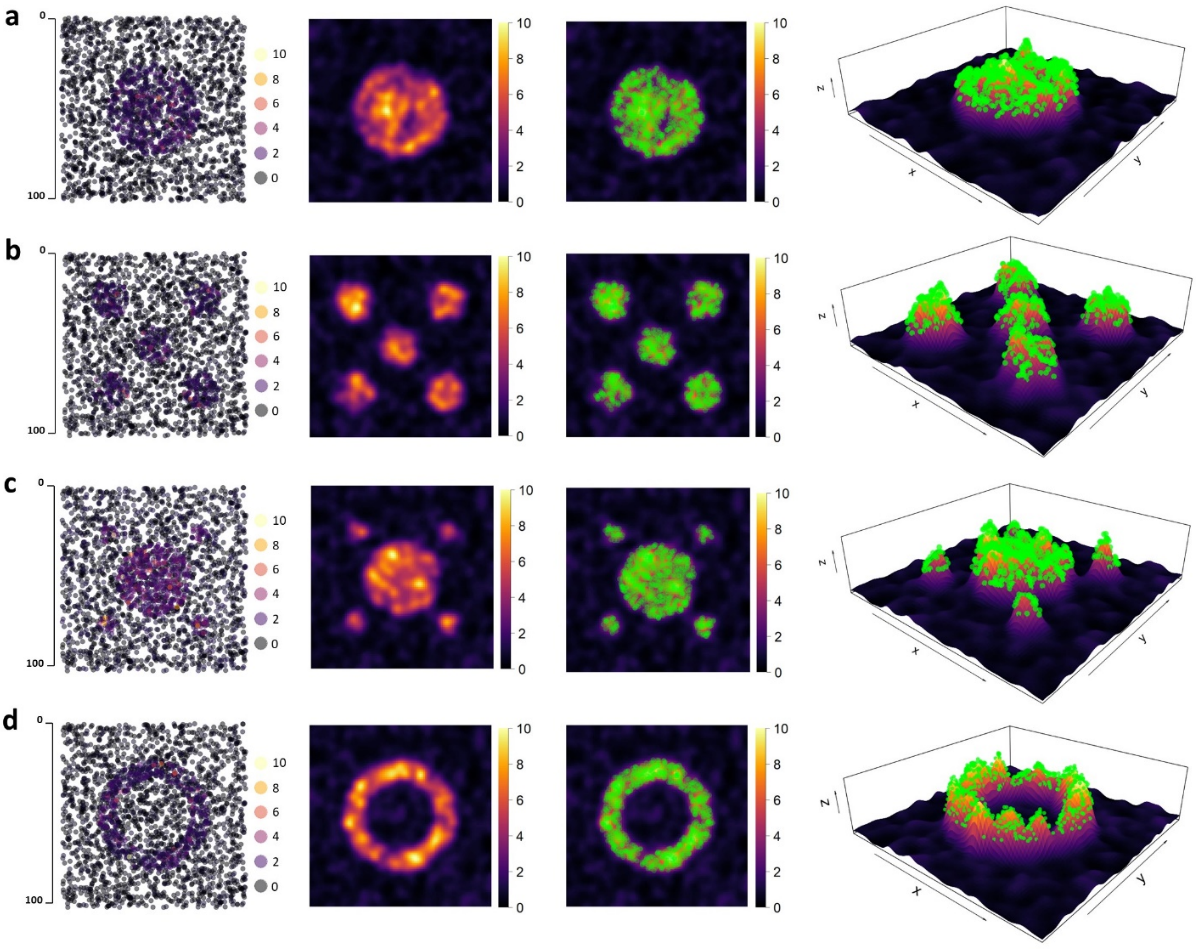

Supplementary Fig. 14. Molecular Probabilistic Maps (MPMs) correctly assign significant relative spatial abundance/ deficiency in simulated data. For all four spatial ground-truths, intensity values were sampled from above and below the upper quartile of the empirical intensities of a MALDI-TOF-MSI measurement of a human gastrointestinal stromal tumor (GIST) tissue sample (**Supplementary Fig. 20**) at *m/z* 494.2662 (imatinib $[M+H]^+$; SNR ≥ 3) for the simulated high MOI abundance and background areas, respectively. Spatial parameters of the simulated SPPs are similar to the ones described in **Fig. 2b** and **Supplementary Fig. 13**. The MPM method was able to identify points exhibiting significant relative spatial abundance (green points on density and surface plots) in accordance with the corresponding ground-truth.

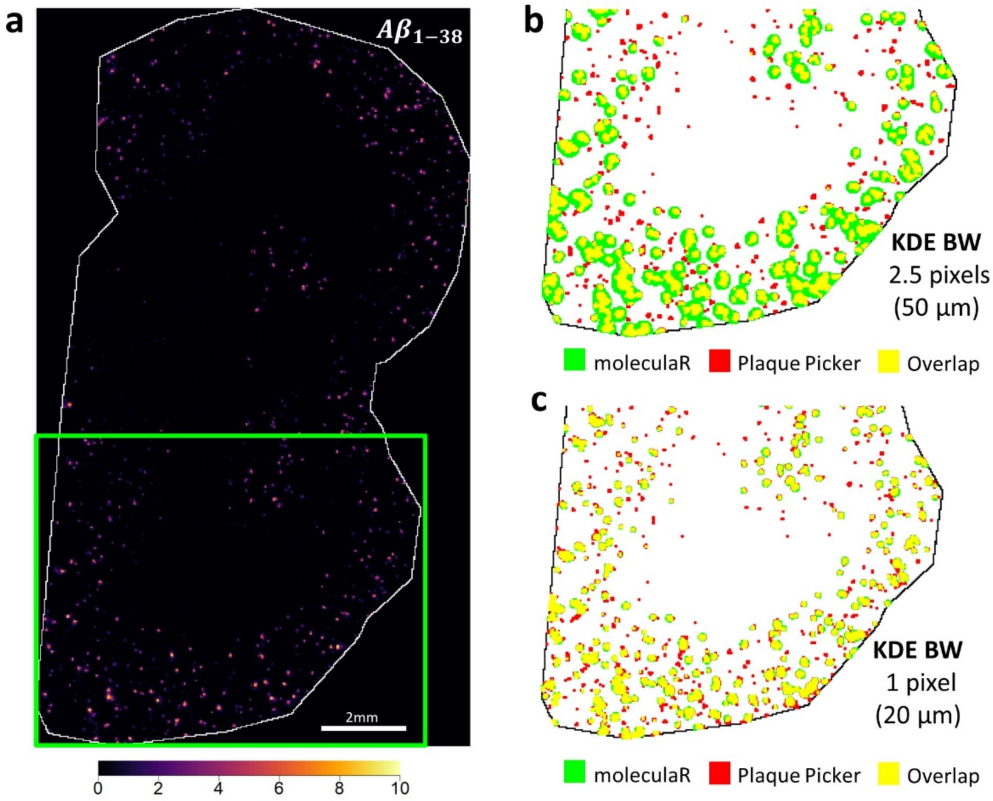

Supplementary Fig. 15. *moleculaR*'s MPMs compared to computational MSI data analysis with the previously published *Plaque Picker*[3] method that was specifically designed for detecting sparsely distributed signals of the Alzheimer's Disease-associated $A\beta_{1\text{-}38}$ amyloid peptide. Here, a previously published MALDI-TOF-MSI example of an APP NL-G-F mouse model is shown. **a)** Intensity distribution of amyloid plaques/deposits as defined by presence of $A\beta_{1\text{-}38}$ at *m/z* 4060.5 (raster size of 20 µm). **b)** Out-of-the-box plaque detection via *moleculaR* (green spots) with a data-inferred KDE bandwidth of 2.5 pixels (50 µm). MPM computation correctly identifies pockets of $A\beta_{1\text{-}38}$ plaques identified as such by *Plaque Picker* (red spots), but tends to not identify small, single-pixel-sized ones. **c**) *moleculaR* enables the custom assignment of KDE bandwidth based on a theoretical size consideration. While $A\beta_{1\text{-}38}$ plaques are known to vary in shape and size. Taking this into consideration, the KDE bandwidth was assigned to a single pixel length, corresponding to the smallest possible scale of 20 µm (i.e. the spatial resolution limit of the measurement) or 400 μm^2 pixel area. The resulting hotspot contours provide a more precise overlap to the spots declared as $A\beta_{1\text{-}38}$ plaques by *Plaque Picker*.

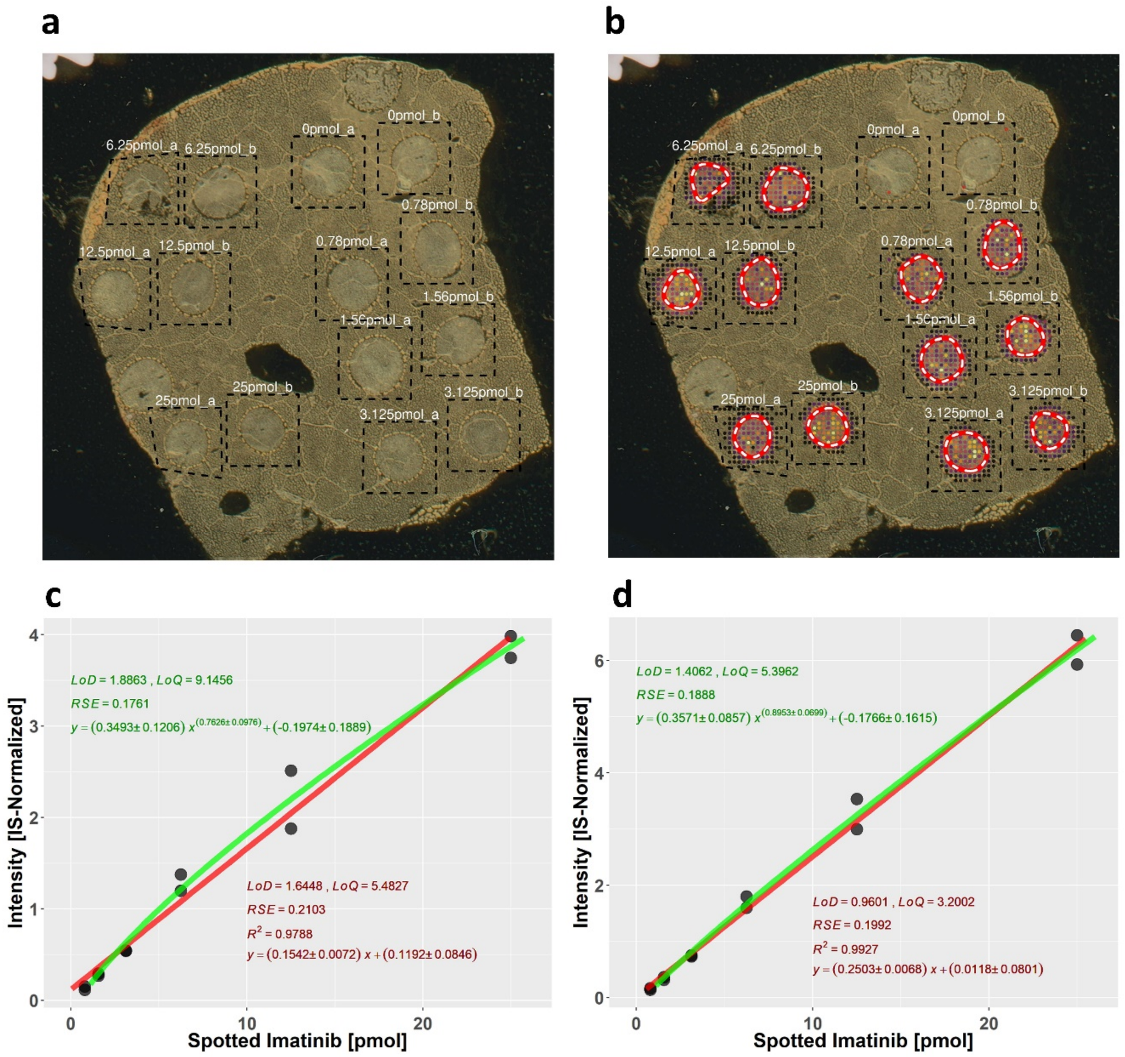

Supplementary Fig. 16. MPMs applied on an imatinib dilution series (25, 12.5, 6.25, 3.125, 1.56, 0.78 pmol and a blank control) spotted onto porcine liver tissue and assessed by MALDI-FTICR-quantitative MSI as previously published [5]. **a)** Optical image of the tissue section showing the measurements spots of the imatinib dilution series. **b)** MPM hotspot contours superimposed on the dilution measurement spots. Raster points indicate pixels where the imatinib signal was detected. MPMs were generated for each spot individually (i.e. one spot at a time). **c)** Curve fitting for the dilution series using the entire measurement area (all pixels that imatinib $[M+H]^+$ was detected in) and applying the conventional uniform mass window weighting. Both linear (red) and non-linear (green) fitted calibration curves are shown. **d)** Linear and non-linear calibration curve fitting using the Gaussian mass window weighting and considering only imatinib signals within the calculated MPM hotspot contours. Compared to **c**, dilution series data points showed higher linearity, as suggested by higher R^2 of the linear curve fit and x-exponent of the non-linear curve fit, respectively. This initial data suggests that the use of the MPM workflow in quantitative MSI may be worth exploring more systematically. Source data are provided as a Source Data file.

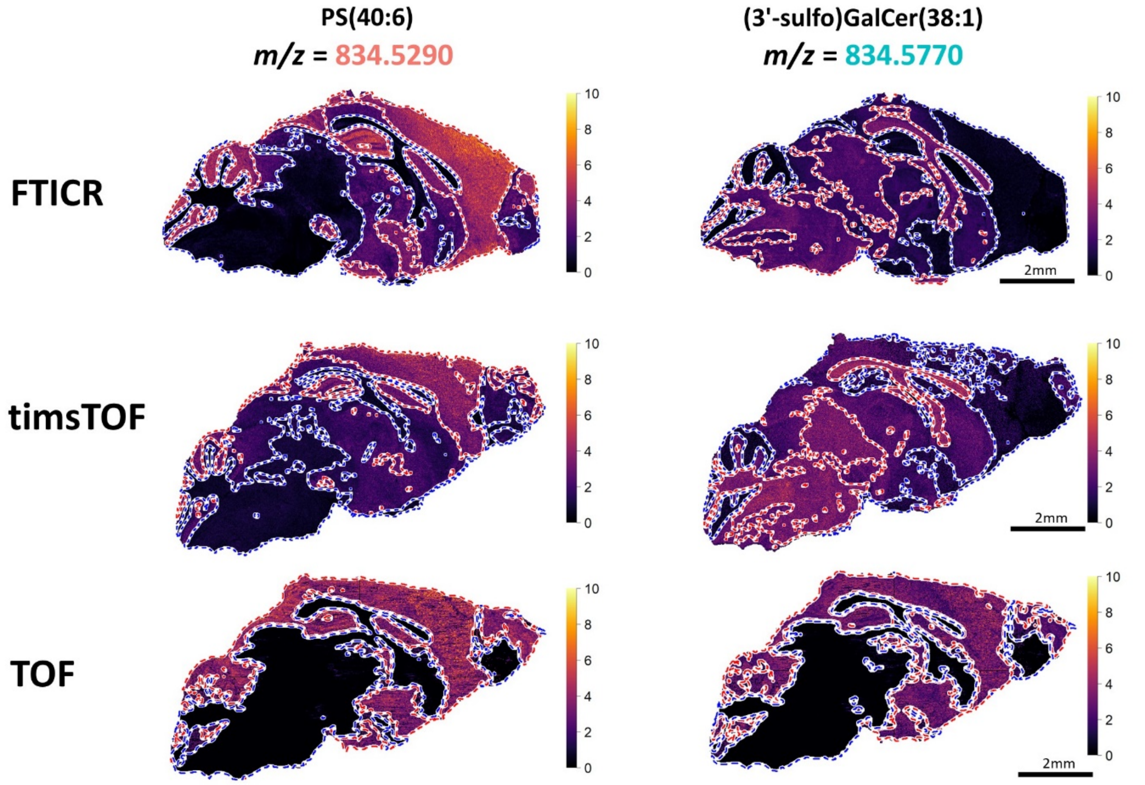

Supplementary Fig. 17. Cross-platform evaluation of MPMs of the exemplary MOIs of **Supplementary Fig. 6, Supplementary Fig. 7** and **Supplementary Fig. 8,** phosphatidylserine PS(40:6)[M-H]$^-$ (MOI1; *m/z* 834.5290; FDR ≤0.05; light red) and C20:0 sulfatide (3'-sulfo)GalCer(38:1)[M-H]$^-$ (MOI2; *m/z* 834.5770; FDR ≤0.10; turquoise), shown on MALDI-FTICR MSI, timsTOF and TOF data of a sagittal mouse brain tissue section. The hotspot contours of FTICR and timsTOF, but not TOF, are able to differentiate MOI1 and MOI2 due to the limited resolving power of the MALDI-TOF MSI measurement.

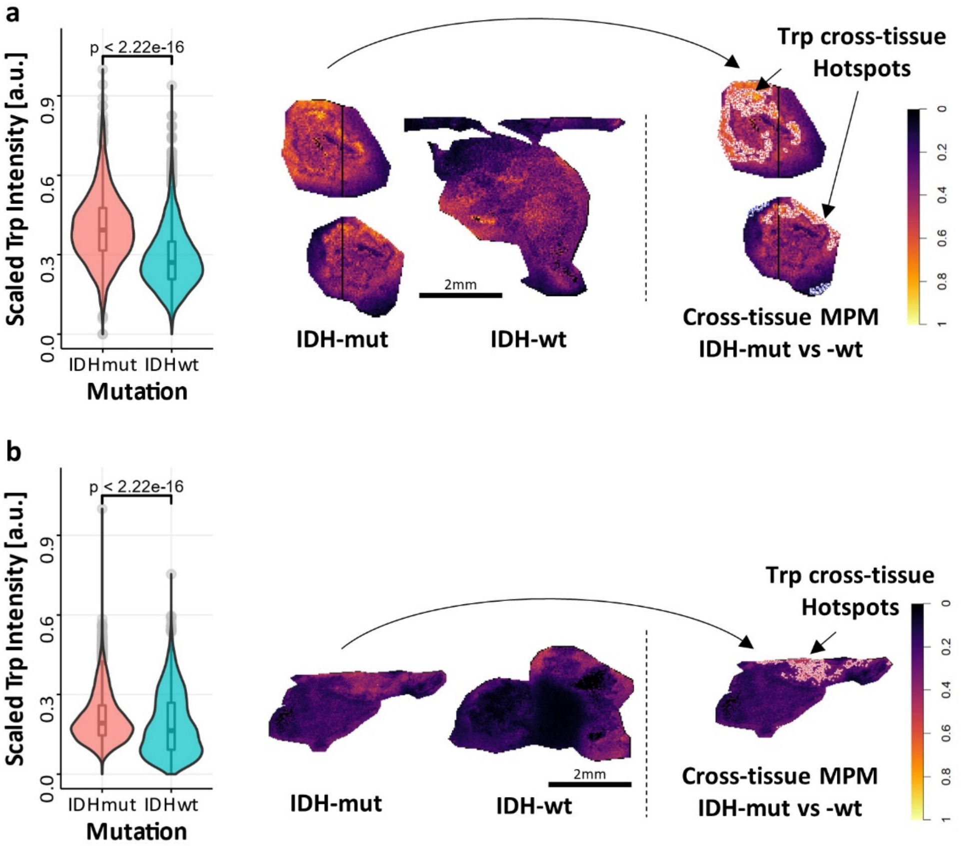

Supplementary Fig. 18. Cross-tissue molecular probabilistic maps (CT-MPMs) enable spatially-aware cross-tissue comparison of tryptophan $[Trp-H]^-$ distribution in isocitrate dehydrogenase (IDH)-mutant GB as test tissue compared to IDH-wild type GB as reference tissue. **a** and **b** show two additional human tissue samples, which, together with the samples of **Fig. 3e**, we reported in Friedrich et al.[4] to have significantly higher tryptophan levels in IDH-mutant GB compared to IDH-wild type GB. Cross-tissue MPMs provide the advantage of spatial statistical testing where normally only signal intensities are used for statistical comparisons (e.g., box/violin plots; $P < 2.22 \times 10^{-16}$ for **a** and **b**; two-sided Wilcoxon rank sum test; *n*=1993 and *n*=5004 detected signals in **a** and *n*=9148 and *n*=4872 detected signals in **b** for IDHmut and IDHwt, respectively) that disregard the spatial localizations of MOIs under study. This is achieved by first finding hotspots/ coldspots, i.e. areas of significant relative spatial abundance/deficiency as shown in **Fig. 2a.** Then all pixel intensities of the test tissue (here, IDH-mut GB) are tested against the empirical cumulative distribution function inferred from the pixel intensities of the reference tissue (here, IDH-wt GB). Test tissue intensities which reject **both** the spatial null (= MOI is spatially randomly distributed) and Test-vs-Reference intensity distributions null hypotheses are designated as having significant cross-tissue relative abundance/deficiency. Please, note that the areas of significantly increased spatial abundance (= MPM hotspot contours) of Trp in the IDH-mutant cases (compared to IDH-wt cases) are spatially much more restricted than a user may have guessed based on Trp ion images for the IDH-mutant cases alone. Boxplots indicate median (middle line), 25th, 75th percentile (box), and whiskers which extend to the most extreme data point which is no more than 1.5 times the length of the box away from the box. Source data are provided as a Source Data file.

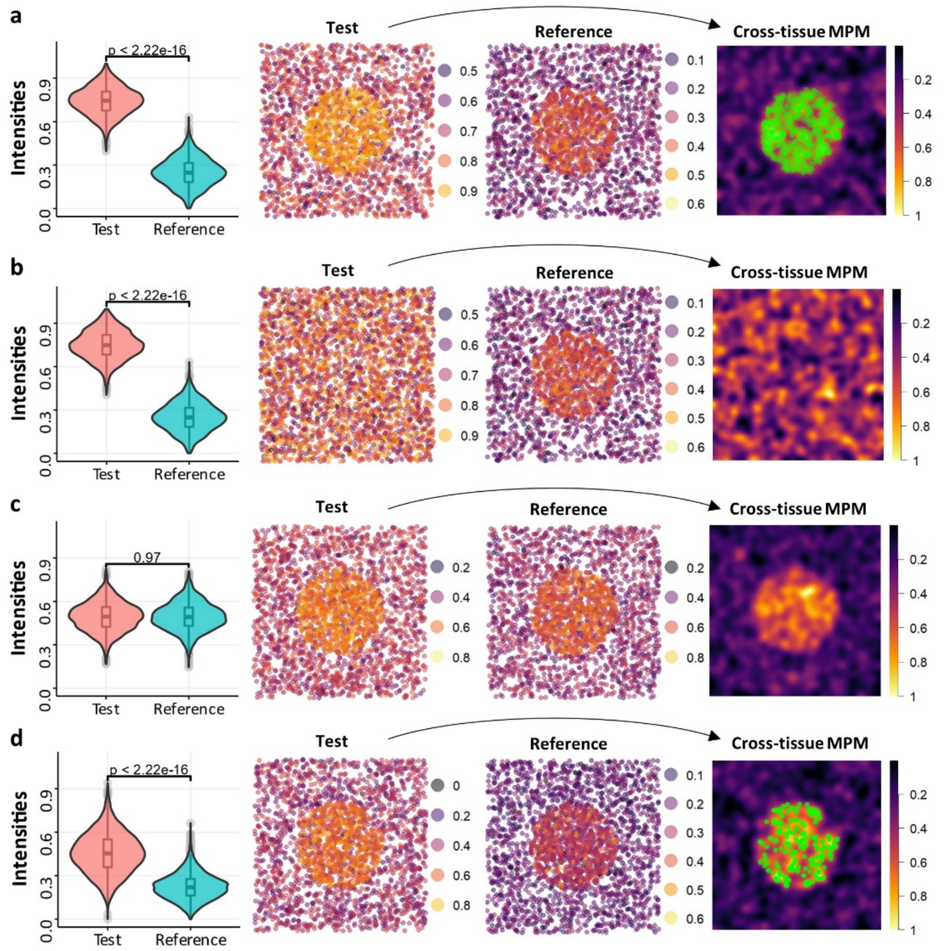

Supplementary Fig. 19. Computation of cross-tissue molecular probabilistic maps (CT-MPMs) based on a simulated spatial point patterns (SPP) in "Test" versus "Reference" datasets. A simulated high MOI abundance area (a spatial circular structure; see **Supplementary Fig. 11a** and **Supplementary Fig. 13a**) in the "Test" -SPP is either present (**a**, **c**, **d**) or absent (**b**). Moreover, a statistically significant difference between the simulated MOI intensities of "Test"- and "Reference"-SPPs is either present (**a**, **b**, **d**; two-sided Wilcoxon rank sum test; $P < 2.22\times10^{-16}$; for **a** Test n=2594 and Reference n=2579; for **b** Test n=2992 and Reference n=2579; for **d** Test n=2594 and Reference n=2614) or absent (**c**; two-sided Wilcoxon rank sum test; P=0.97; Test n=2634 and Reference n=2590). "Test" and "Reference" SPP intensities in **a** and **b** were drawn from normal distributions with μ of 0.75 and 0.25 (σ = 0.1 for both), respectively. In **c,** they were drawn from the same normal distribution with μ = 0.5 and σ = 0.1, and in **d** from the empirical intensities of a MALDI-FTICR-MSI measurement of IDH-mut ("Test") and IDH-wt ("Reference") human GB tissue sample (**Fig. 3e**) at *m/z* 203.0815 ($[Trp\text{-}H]^-$). Simulated SPPs were generated as described in **Supplementary Fig. 13a**. "Test" SPP points' intensities, for which **both** the spatial null hypothesis (i.e. MOI intensities follow a complete spatial randomness model) **and** the Test-vs-Reference intensity distributions null hypothesis are rejected, are labeled with green points for having significant cross-tissue relative abundance (right-most panels of **a** and **d**). Despite the statistically significant difference in MOI intensity distributions in **b**, no significant cross-tissue relative abundance was detected in the corresponding "Test" SPP, because it did not exhibit a spatial trend (i.e. it consequently did not reject the spatial null hypothesis). On the other hand, despite the clear presence of a non-random spatial pattern in **c**, no significant cross-tissue relative abundance was detected because MOI intensities of the "Test" and "Reference" SPPs were drawn from the same intensity distribution. Boxplots indicate median (middle line), 25th, 75th percentile (box), and whiskers which extend to the most extreme data point which is no more than 1.5 times the length of the box away from the box. Source data are provided as a Source Data file.

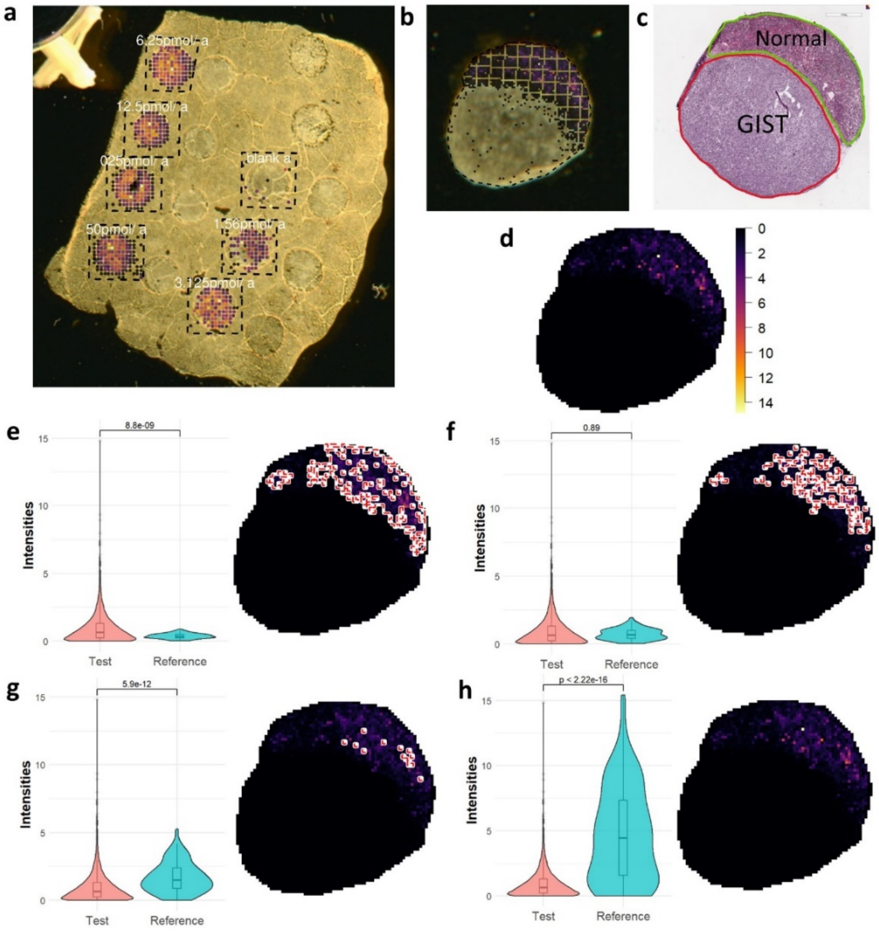

Supplementary Fig. 20. Cross-tissue molecular probabilistic Map (CT-MPM) was used to spatially localize areas of significant cross-tissue relative abundance of imatinib in a gastrointestinal stromal tumor (GIST) tissue sample when compared against a series of imatinib dilution spots in previously reported MALDI-TOF-MSI data[5]. **a**) Optical image of a porcine tissue section where the imatinib dilution series (25, 12.5, 6.25, 3.125, 1.56, 0.78 pmol and a blank control) was spotted. MALDI-TOF-MSI measurement regions are indicated by dashed polygons and pixels with imatinib signal (SNR ≥ 3; *m/z* 494.2662; $[M+H]^+$) are superimposed. **b**) Optical image of a metastatic GIST tissue sample with superimposed imatinib signal (SNR≥3). **c**) Histopathology-annotated hematoxylin and eosin (H&E) image of the resected GIST tissue (**b**) showing both tumor (red) and non-tumor (normal; green) tissue. **d**) An intensity image of imatinib (*m/z* 494.2662; $[M+H]^+$) in the GIST tissue sample (**b**). Mean imatinib content in the sample tissue was previously reported to be 7.78 pmol (95% CI 7.28, 8.46 pmol) and 7.81 pmol (95% CI 7.63, 7.99 pmol) based on MALDI-TOF-MSI and UPLC-ESI-QTOF-MS quantification, respectively [5]. Panels **e-h** illustrate CT-MPMs computed for the tissue sample in **d** compared against imatinib dilution series spots of 3.125 (**e**), 6.25 (**f**), 12.5 (**g**) and 25 pmol (**h**), respectively. Accompanying box/violin plots show the differential distribution of imatinib signal intensities between the Test tissue (i.e. **d**; *n*=1468) and the corresponding Reference dilution spots of **e** (*n*=104; P=8.80×10^{-9}), **f** (*n*=114; P=0.89), **g** (*n*=88; P=5.94×10^{-12}), and **h** (*n*=118; P < 2.22×10^{-16}; a two-sided Wilcoxon rank sum test has been used for all panels). CT-MPMs are able to spatially localize tissue areas with significant cross-tissue relative abundance of imatinib, which i) mainly concentrate in the non-tumor tissue part, ii) the higher the imatinib intensity in the reference (i.e. in spot of the dilution series in **a**), the smaller the area in the tumor tissue that is labeled as "significant presence" (green dots) in the CT-MPMs. Boxplots indicate median (middle line), 25th, 75th percentile (box) and whiskers which extend to the most extreme data point which is no more than 1.5 times the length of the box away from the box. Source data are provided as a Source Data file.

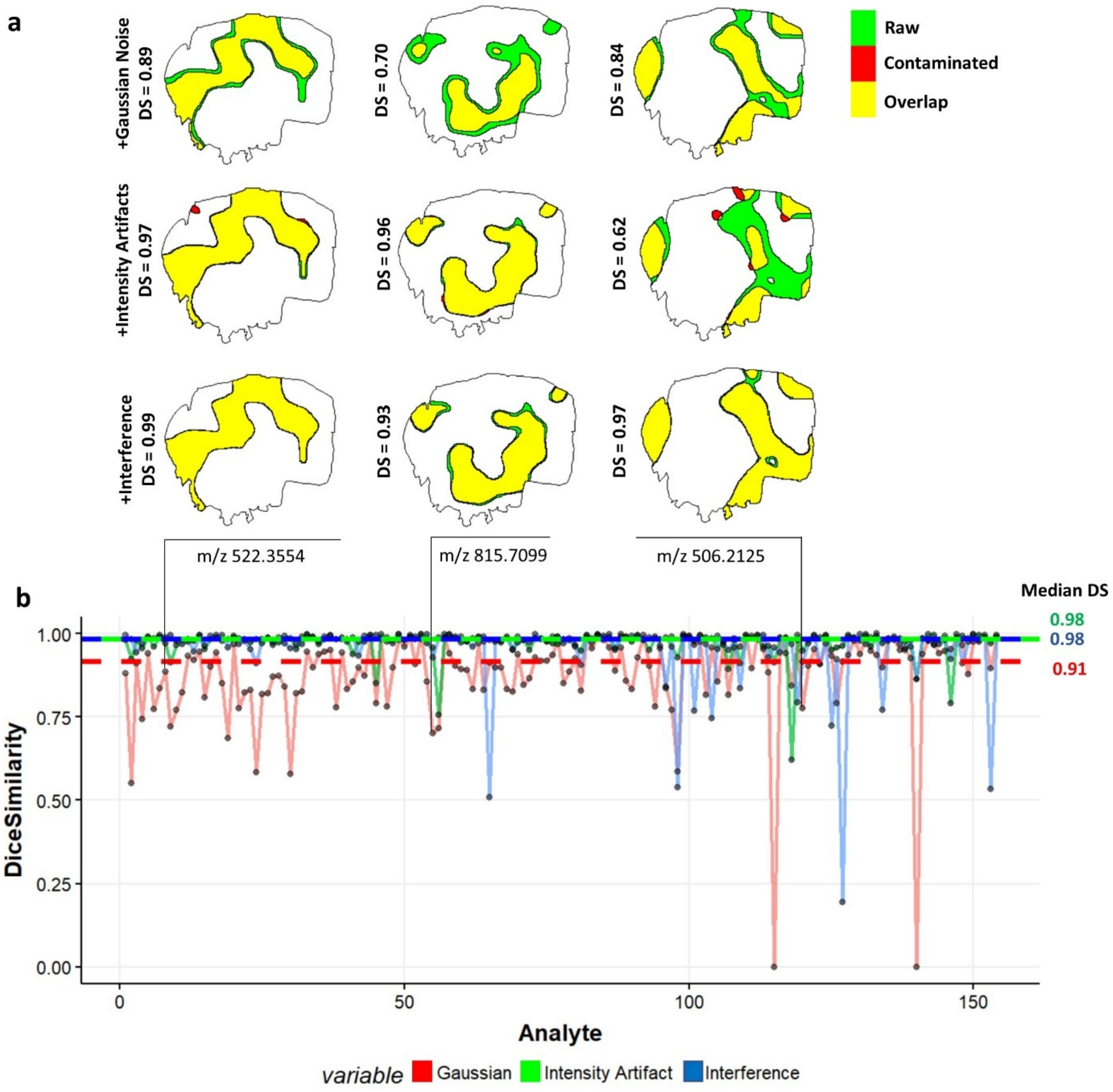

Supplementary Fig. 21. Dice similarity coefficients (DSC) computed for MOI estimated hotspot contours between raw data against artificially contaminated (with different types of noise) data of 142 MPMs of MOIs identified in Metaspace with FDR ≤0.2 in positive ion mode. Noise was artificially added according to the three noise types presented in **Fig. 4ab**; Gaussian noise added to MOI (first row of **a**; red curve in **b**), presence of abnormally high-intensity peak artefacts (second row of **a**; green curve in **b**) and added interfering noise occurring within the span of the theoretical Gaussian envelop specific for MOI ($2\sigma_G$ away from MOI *m/z*; third row of **a**; blue curve in **b**). Median Dice similarity coefficients were 0.91 (dashed red line), 0.98 (dashed green line) and 0.98 (dashed blue line) for the contamination sources Gaussian noise, intensity artefacts and interference, respectively. Source data are provided as a Source Data file.

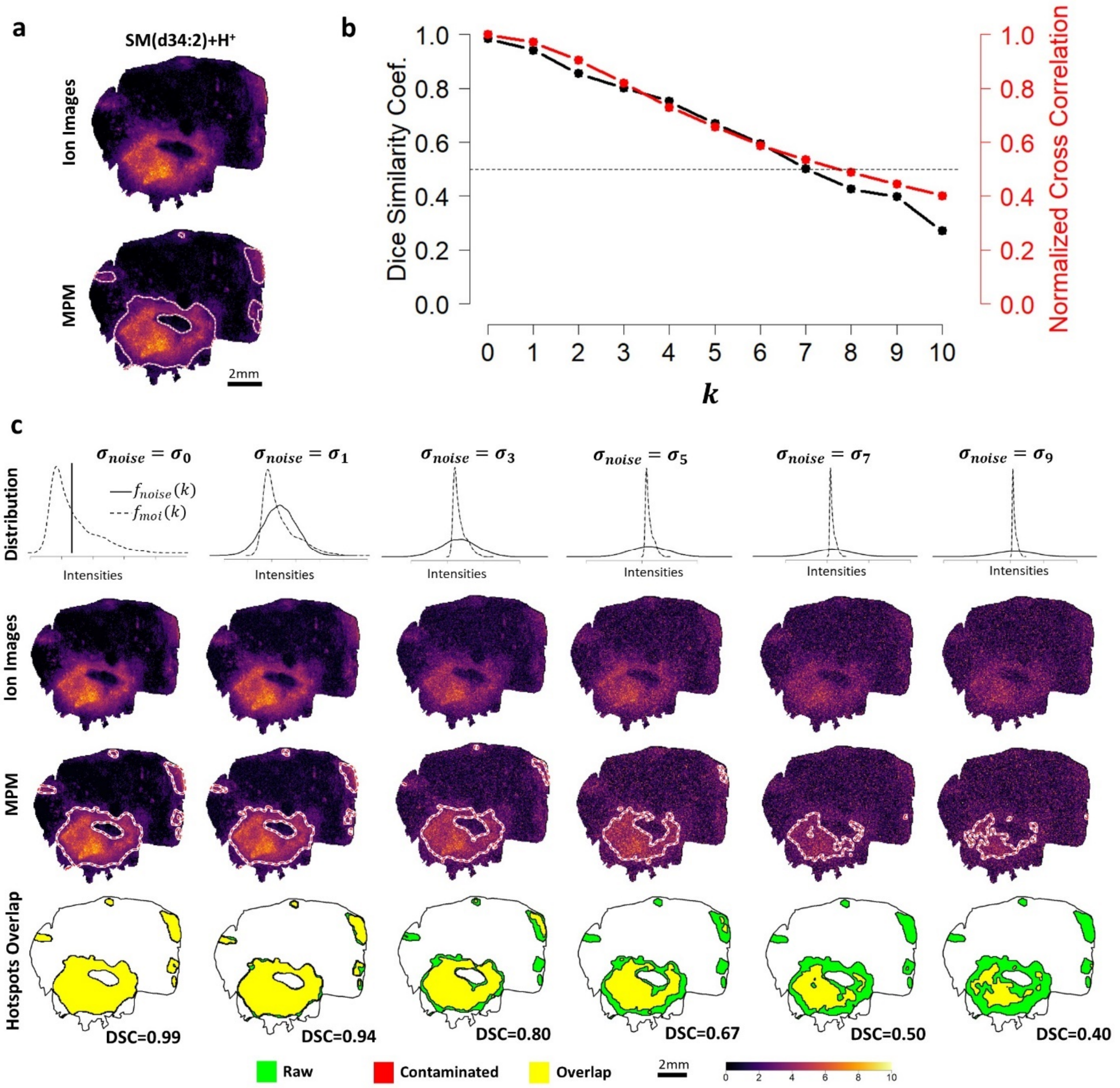

Supplementary Fig. 22. Evaluating the robustness of MPM against artificially added noise spiked at MOI *m/z*. **a)** Ion image and MPM of an example MOI, sphingomyelin SM(d34:2)[M+H]$^+$ (FDR ≤ 0.1). **b)** Gaussian noise was added iteratively according to the scheme in **Fig. 4ab** to the peak signal of **a** with varying $\sigma_{\text{noise}} = \sigma_k \ (k = 0 \dots 10)$, where $\sigma_0 = \sqrt{\mu_{\text{MOI}}}$ and $\sigma_k = k\ \sigma_{\text{MOI}}$ for $k = 1 \dots 10$ and σ_{MOI} and μ_{MOI} are the standard deviation and mean of the signal intensities of MOI. Note that for $k = 0$, the resulting noise is similar to Poisson noise with $\lambda_{\text{Poisson}} = \mu_{\text{MOI}} \gg 1000$. At each iteration the Dice similarity coefficient (DSC) was computed between the estimated MPM hotspot contour of the raw and artificially contaminated data (black solid curve) while also computing the normalized cross correlation (NCC) similarity metric between the raw and artificially contaminated images (red solid curve). **c)** Visual illustrations of MOI signal intensity distribution $f_{MOI}(k)$ relative to the added noise distribution $f_{noise}(k)$ at each noise iteration (first row), corresponding ion images (second row), calculated MPM hotspot contours (third row) and hotspot contours overlap between raw and artificially contaminated data (bottom row). MPM hotspot contours are able to withstand Gaussian noise with σ_n up to 4 times σ_{MOI} retaining hotspot contours overlap of above 0.75 DSC, all this while observing clear visual degradation of the corresponding ion images which is also mirrored in their computed NCC relative to the raw ion image. Source data are provided as a Source Data file.

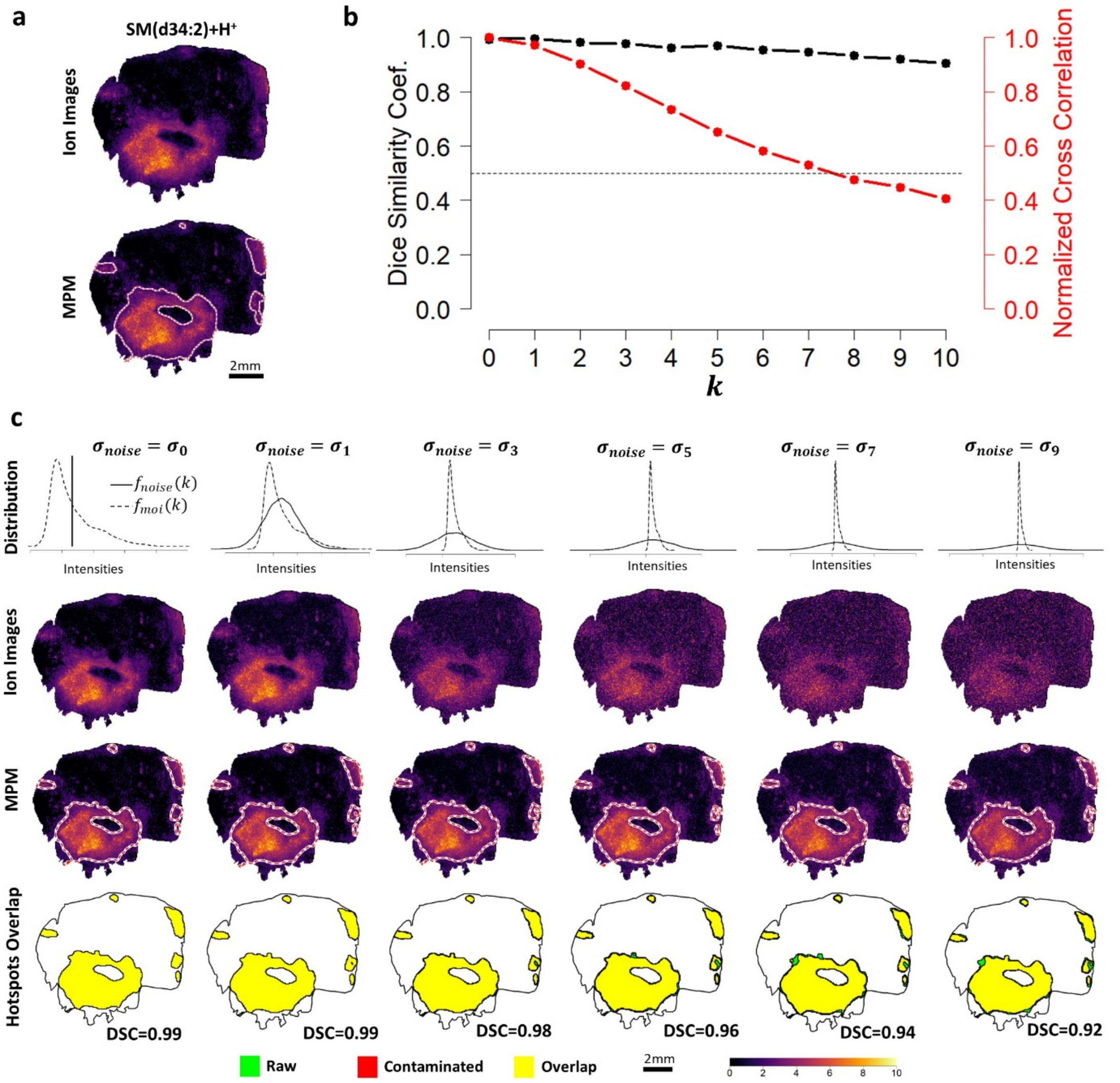

Supplementary Fig. 23. Evaluating the robustness of MPM against artificially added **interfering** noise spiked in the vicinity (i.e. $2\sigma_G$ away) of *m/z* MOI. **a)** Ion image and MPM of an example MOI, sphingomyelin SM(d34:2)[M+H]$^+$ (FDR ≤ 0.1). **b)** Artificial Gaussian noise was added iteratively according to the scheme in **Fig. 4ab** at $2\sigma_G$ away from the peak signal of **a** (i.e. at *m/z* MOI + $2\sigma_G$) with varying $\sigma_{\text{noise}} = \sigma_k$ $(k = 0 \ldots 10)$, where $\sigma_0 = \sqrt{\mu_{\text{MOI}}}$ and $\sigma_k = k\,\sigma_{\text{MOI}}$ for $k = 1 \ldots 10$ and σ_{MOI} and μ_{MOI} are the standard deviation and mean of the signal intensities of MOI. Note that for $k = 0$, the resulting noise is similar to Poisson noise with $\lambda_{\text{Poisson}} = \mu_{\text{MOI}} \gg 1000$. At each iteration the Dice similarity coefficient (DSC) was computed between the estimated MPM hotspot contours of the raw and artificially contaminated data (black solid curve) while also computing the normalized cross correlation (NCC) similarity metric between the raw and artificially contaminated ion images (red solid curve). **c)** Visual illustrations of MOI signal intensity distribution f_{MOI} *(k)* relative to the added noise distribution f_{noise} *(k)* at each noise iteration (first row), corresponding ion images (second row), calculated MPM hotspot contours (third row) and hotspot contours overlap between raw and artificially contaminated data (fourth row). As a consequence of the Gaussian mass-window weighting, MPM hotspot contours are able to withstand interfering Gaussian noise (spiked at *m/z* MOI + $2\sigma_G$) with σ_n up to 10 times σ_{MOI} retaining hotspot contours overlap of 0.91 DSC, all this while observing clear visual degradation of the corresponding ion images which is also mirrored in their computed NCC relative to the raw ion image. Source data are provided as a Source Data file.

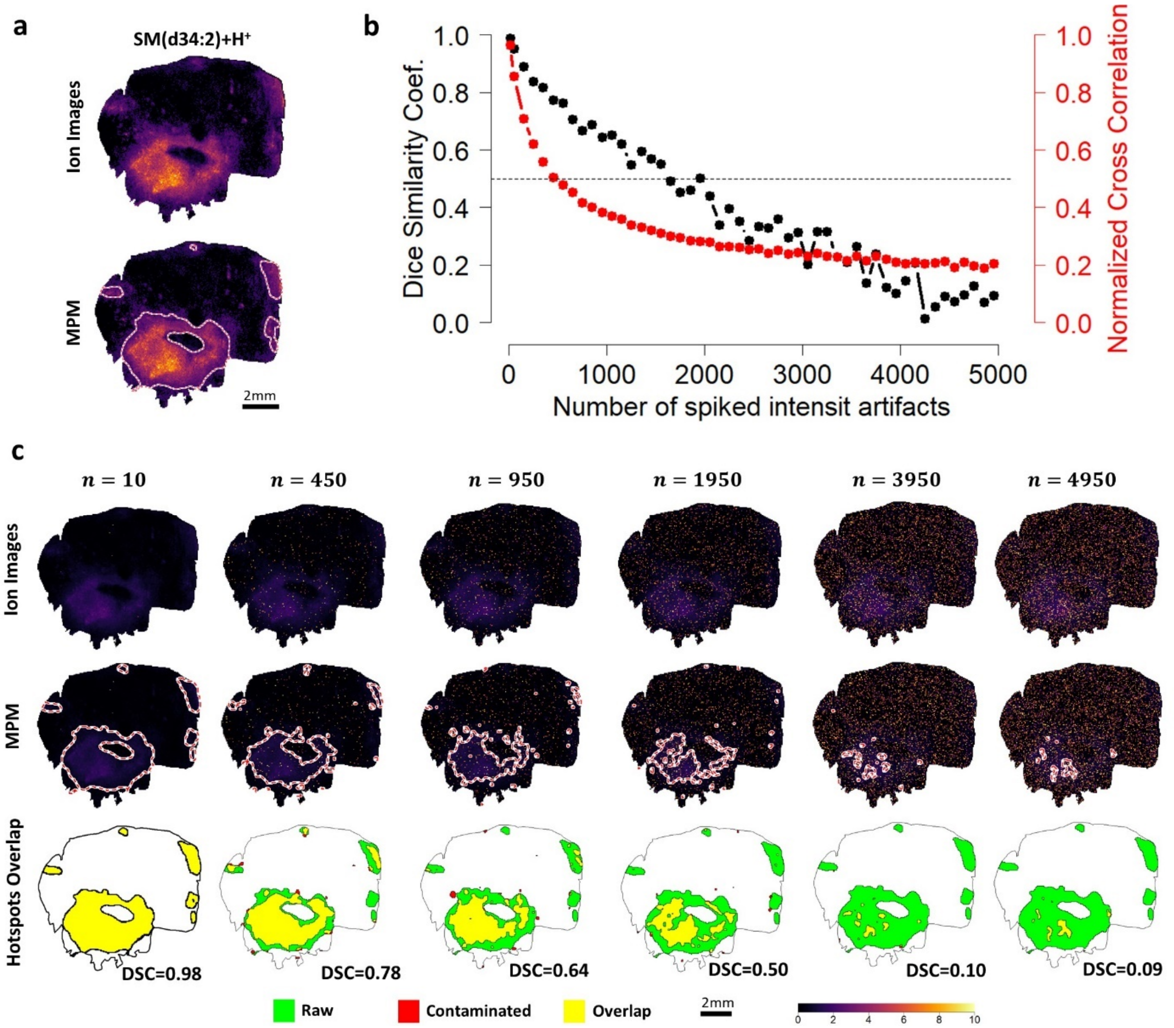

Supplementary Fig. 24. Evaluating the robustness of MPM against artificially added intensity artifacts spiked at MOI *m/z*. **a)** Ion image and MPM of an example MOI, sphingomyelin SM(d34:2)[M+H]$^+$ (FDR ≤ 0.1). **b)** Intensity artifacts was added iteratively according to the scheme in **Fig. 4ab** to the peak signal of **a,** with a varying number of intensity artifacts n (up to n = 5000; ≈ 20% of the total tissue pixels) randomly placed within the tissue window. At each iteration the Dice similarity coefficient (DSC) was computed between the estimated MPM hotspot contour of the raw and artificially contaminated data (black solid curve) while also computing the normalized cross correlation (NCC) between the raw and artificially contaminated images (red solid curve). **c)** Visual illustrations of noise-contaminated ion images (first row), calculated MPM hotspot contours (second row) and hotspot contours overlap between raw and artificially contaminated data (bottom row). MPM hotspot contours are still able to withstand added single-pixel intensity artifacts of n = 450 randomly placed within the tissue window reliably delineating the correct hotspot area and retaining hotspot contours overlap of 0.78 DSC, all this while observing clear visual degradation of the corresponding ion images relative to the raw ion image which is also mirrored in their computed NCC (red curve in **b**) which falls below the 0.5 mark (dashed horizontal line in **b**) for the same n. Source data are provided as a Source Data file.

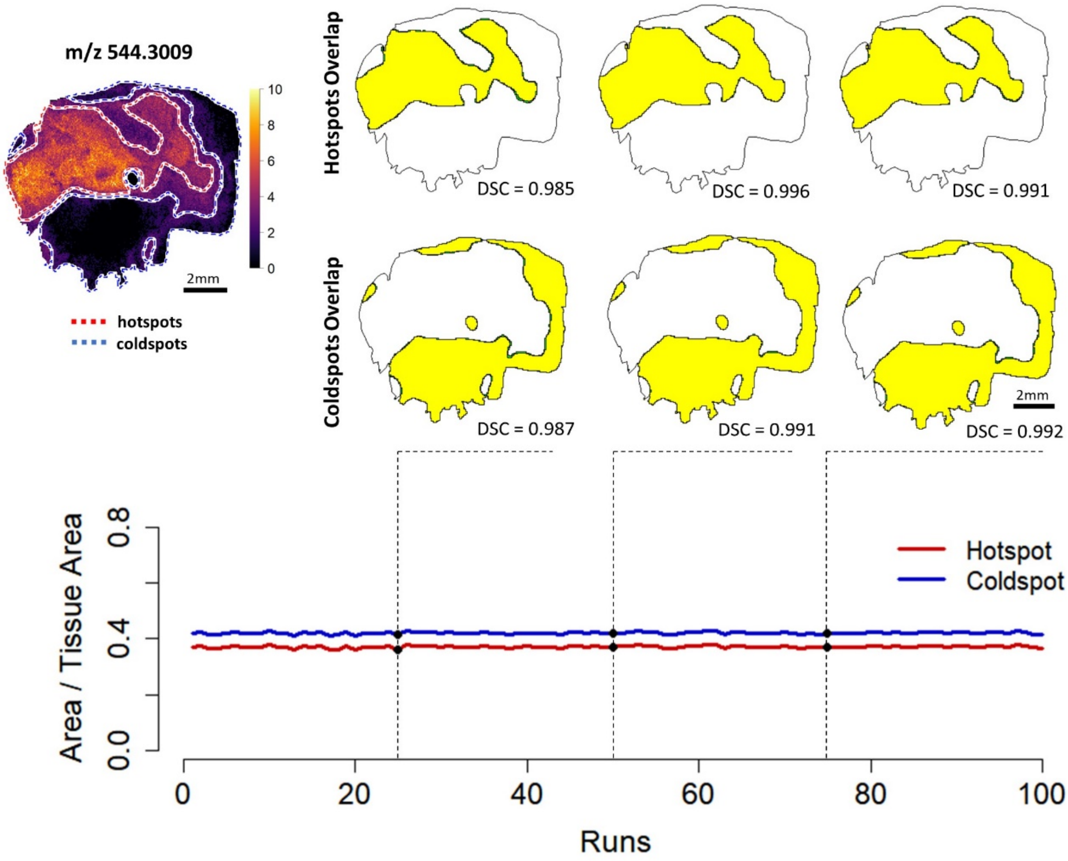

Supplementary Fig. 25. MPM is stable against 100 permutations of the generated complete spatial randomness (CSR) model. MPM was re-computed for the example MOI, PE(20:1)[M+Na]$^+$ (*m/z* 544.3009; FDR ≤ 0.2) of the GB tissue section of **Fig. 3a** 100 times, each time generating a different random permutation of the CSR model. For each iteration, the area of the MOI's MPM hotspot- (red curve) and coldspot (blue curve) contours relative to the total tissue area was computed. The overlap (yellow) between the hotspot and coldspot contour of the 25th, 50th and 75th iterations relative to the hotspot- and coldspot areas of the 1st iteration are shown as examples. Relative areas' mean and standard deviation were 0.3234 and 0.0045 for hotspot contours, respectively, and 0.3771 and 0.0036 for coldspot contours, respectively. DSCs mean and standard deviation between hotspot and coldspot contour areas of each run relative to that of the first run were 0.9883 and 0.0069, respectively, for hotspot contours, and 0.9914 and 0.0046 for coldspot contours, respectively. Source data are provided as a Source Data file.

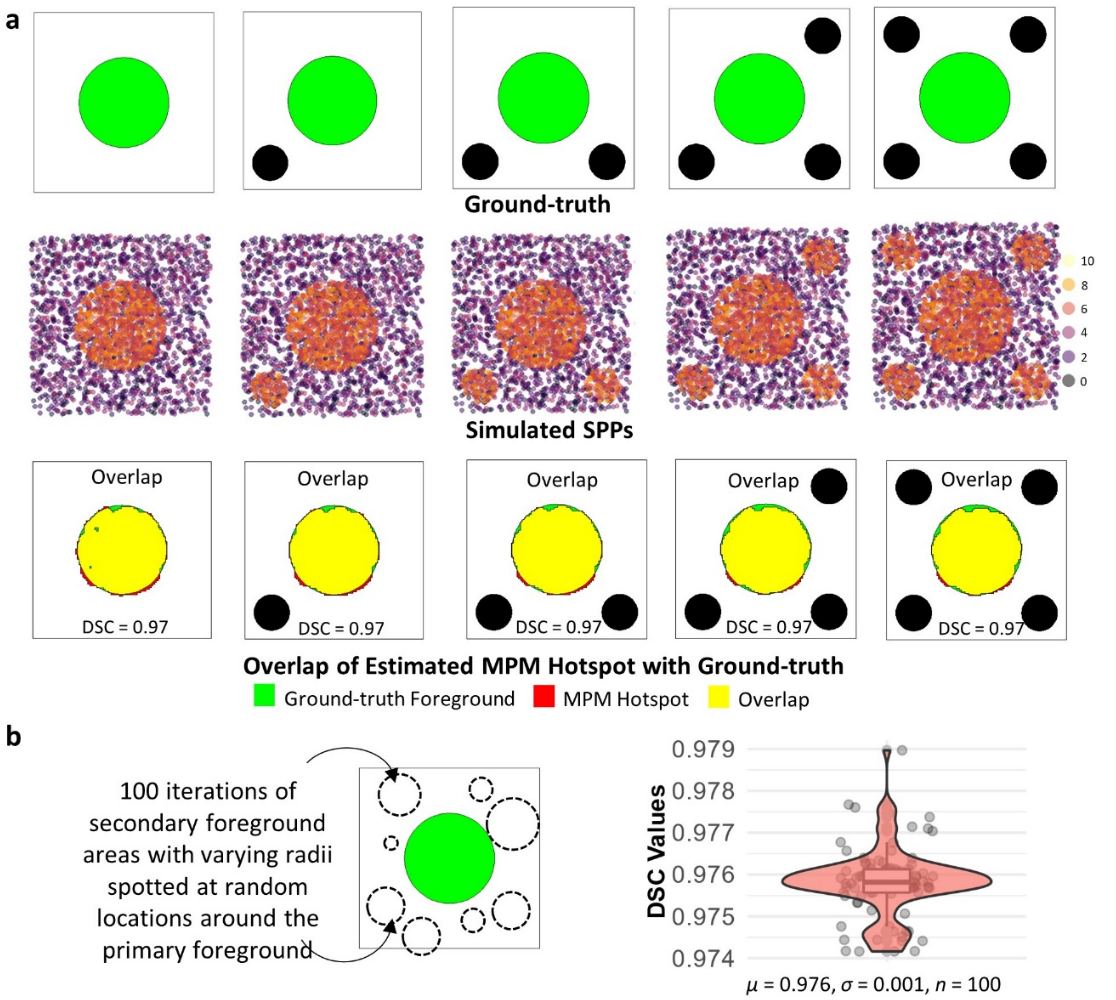

Supplementary Fig. 26. Stability testing of molecular probabilistic maps (MPMs) of an MOI ground truth area (central green filled circle) using simulated data. Here, areas of high-intensity points (black filled circles) were cumulatively (**a**) or iteratively (**b**) added to a ground-truth. A simulated spatial point pattern (SPP) was created based on the ground-truth of **Supplementary Fig. 11a** and **Supplementary Fig. 13a**. **a)** Secondary areas containing high-intensity points (black filled circles) were cumulatively added to an MOI ground truth containing a central area (green filled circles; upper row). Synthetic uniform Poisson SPP simulating an MOI's spatial ground-truth with a circular central area of radius 25 length units (green filled circle) embedded in a background as described in **Supplementary Fig. 13a**. At each step, an MPM was computed for the entire simulated SPP (i.e. central plus added areas), but the Dice similarity coefficient (DSC) was calculated between the green area and its computed MPM hotspot only (i.e. the spotted secondary black areas where excluded from the DSC computation). Note that the MPM hotspot contours of the central green area were unaffected by the cumulative addition of points/areas of high intensity. **b)** Additionally, 100 random ground-truths were created, each time spotting one secondary area (dashed circles) with a randomly chosen radius and position around the central area (green filled circle). At each iteration, MPM and DSC values were computed as described in **a**. Similar to **a**, the computed MPM hotspot contours of the central area were rather unaffected, as indicted by the low variance of the computed DSC values (μ = 0.976, σ = 0.001, n = 100). The Boxplot indicates median (middle line), 25th, 75th percentile (box) and whiskers which extend to the most extreme data point which is no more than 1.5 times the length of the box away from the box. Source data are provided as a Source Data file.

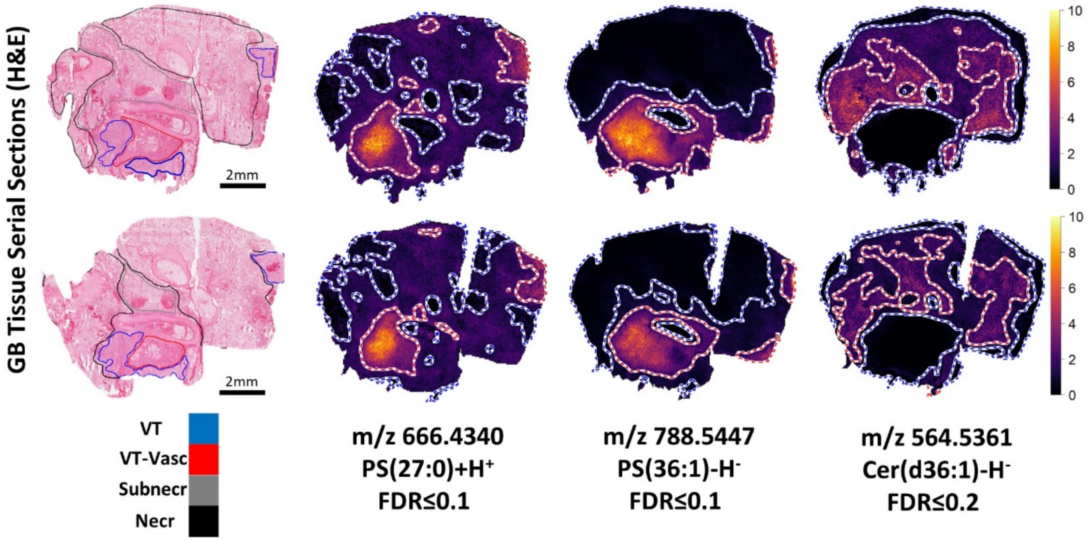

Supplementary Fig. 27. Comparison of the resulting MPMs of three exemplary lipid MOIs shown on two GB tissue serial sections analyzed by MALDI-FTICR-MSI on separate slides (i.e., separate measurement runs) based on raw data (without intensity normalization). MPMs show good agreement across both tissue replicates referenced by the similar localizations for the MOIs' hotspots and coldspots.

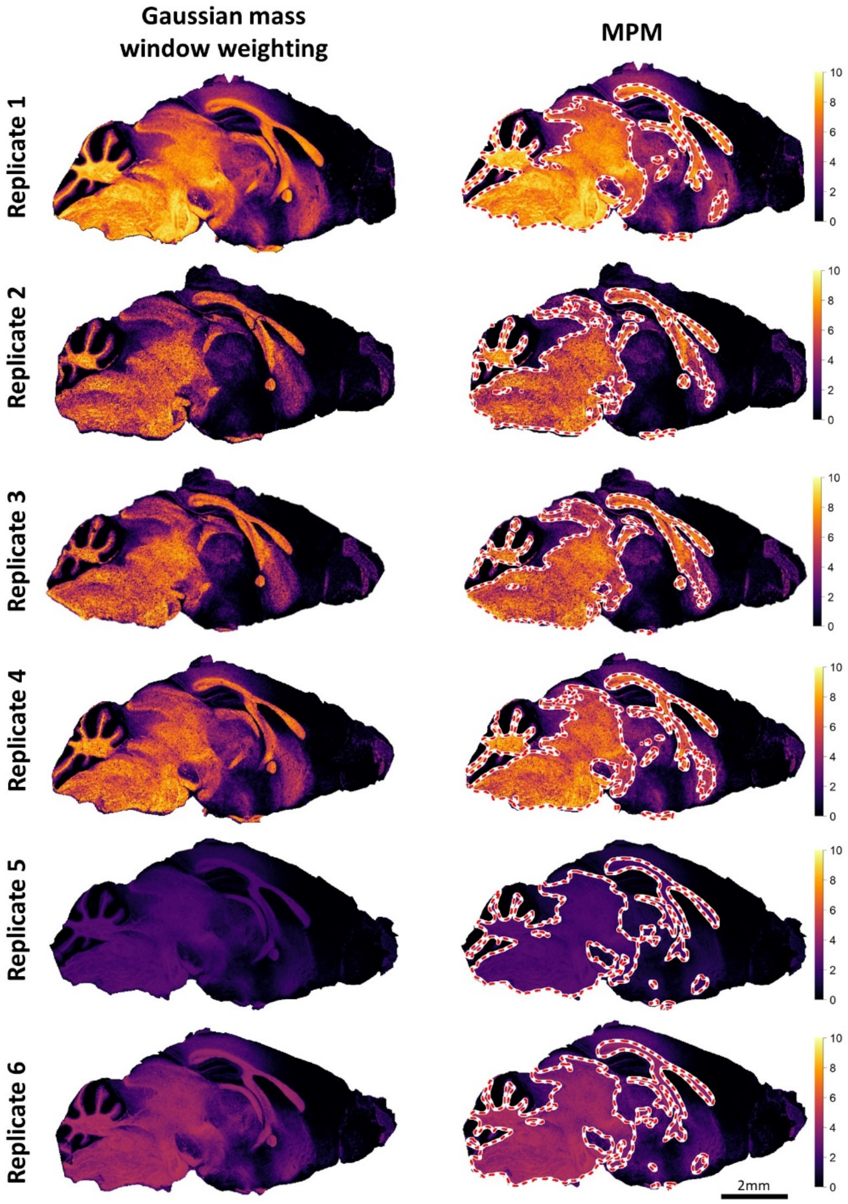

Supplementary Fig. 28. Gaussian mass-window weighted intensity images and the corresponding MPMs of MOI, (3'-sulfo)GalCer(d42:2)$[M-H]^-$ (*m/z* 888.6240; FDR ≤ 0.05) shown on MALDI-FTICR-MSI datasets of six serial sections of sagittal mouse brain tissue. Despite the striking differences in (TIC-normalized) ion intensities across the tissue replicates, MPM hotspot contours show high similarity in their spatial localizations highlighting white matter and mid brain regions.

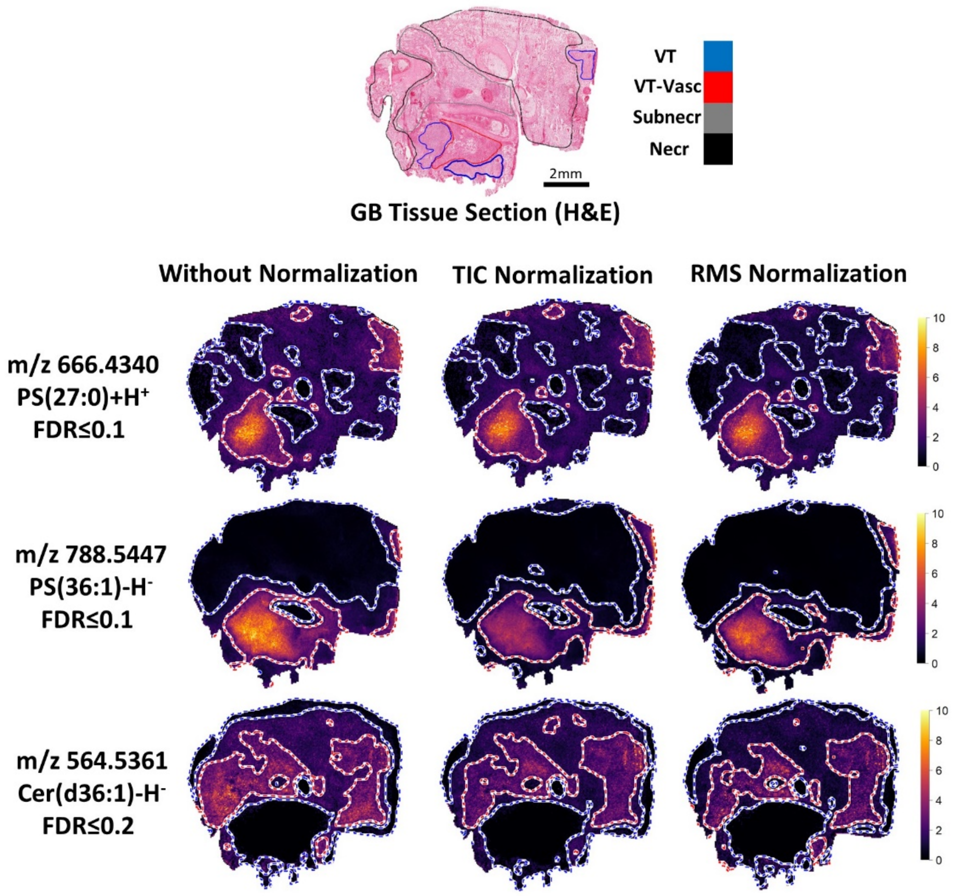

Supplementary Fig. 29. Comparison of the MPMs of three exemplary lipids shown on a GB tissue sections measured with MALDI-FTICR-MSI based on raw data (without intensity normalization), total-ion-count (TIC) normalization and root mean squared (RMS) normalization. MPM hotspot and coldspot contours show good agreement irrespective of the normalization type or the lack thereof.

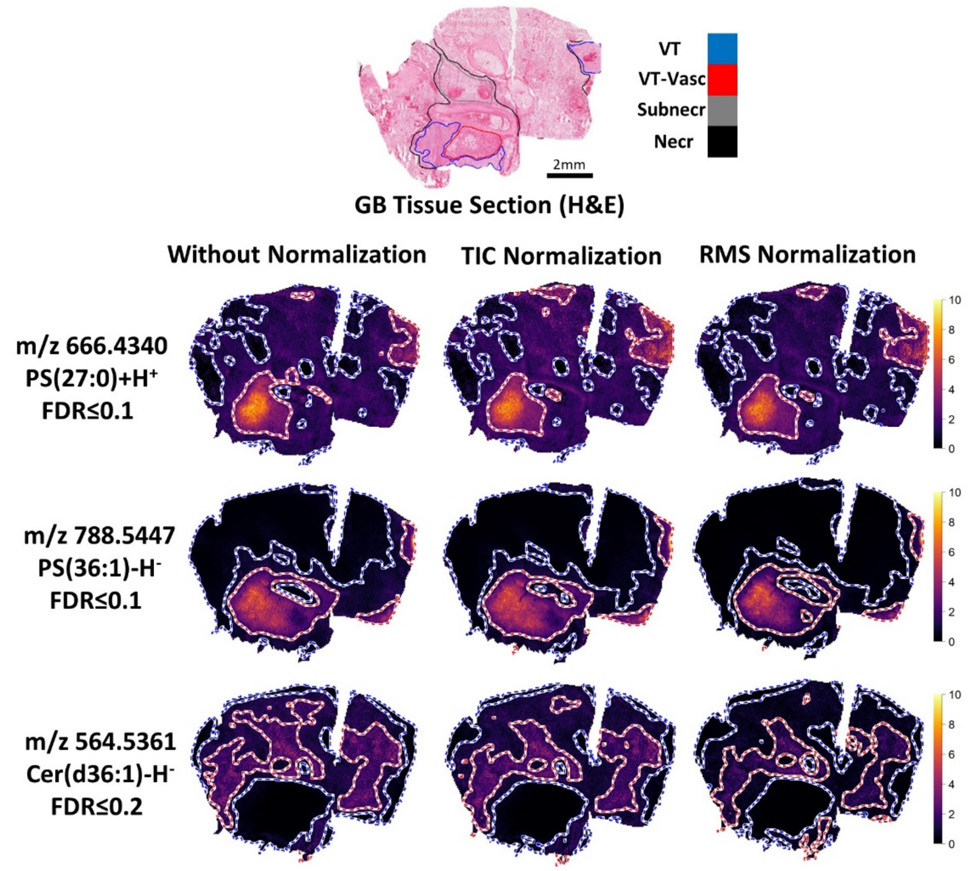

Supplementary Fig. 30. Comparison of the resulting MPMs of three exemplary lipids shown on a GB serial tissue section measured with MALDI-FTICR-MSI based on raw data (without intensity normalization), total-ion-count (TIC) normalization and root mean squared (RMS) normalization. MPM hotspot- and coldspot contours show good agreement irrespective of the normalization type or the lack thereof as also shown for the serial section of **Supplementary Fig. 29.**

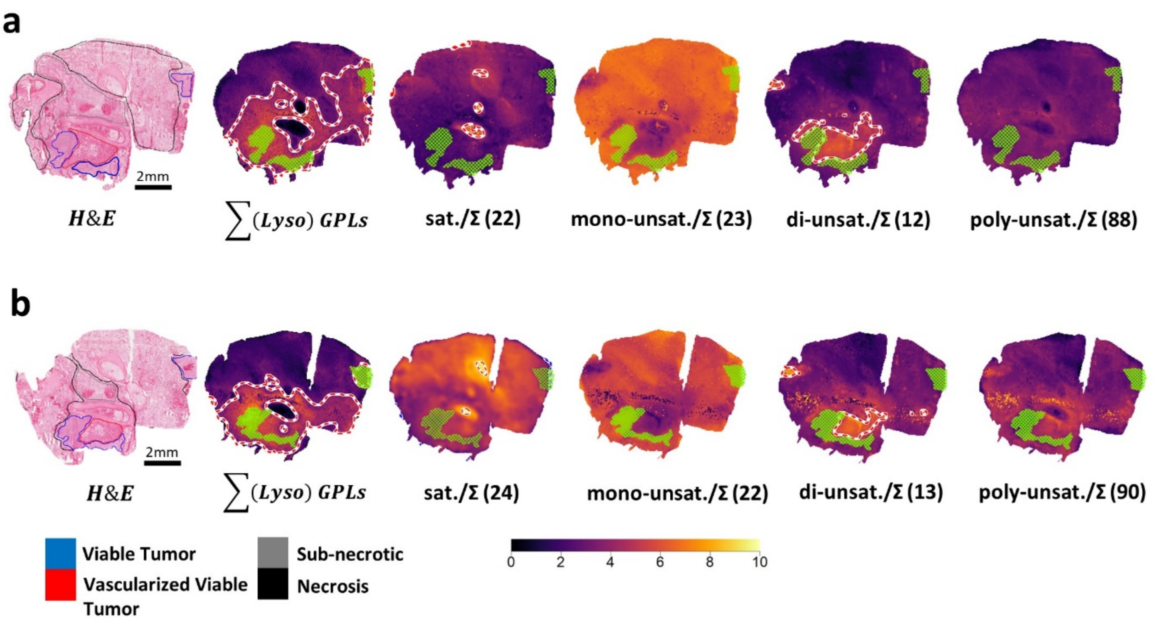

Supplementary Fig. 31. Analysis of the tissue's lipid (un)saturation level of (lyso-)GPLs (PC, LPC, PE, LPE, PS, LPS, PI, LPI) relative to the overall sum Σ(lyso)GPLs (sat: saturated; unsat: unsaturated) for the GB tissue of **Fig. 3a** (**a**) and its serial section (**b**) of **Supplementary Fig. 34**. Numbers in parentheses = Metaspace-verified lipids at FDR ≤ 0.2.

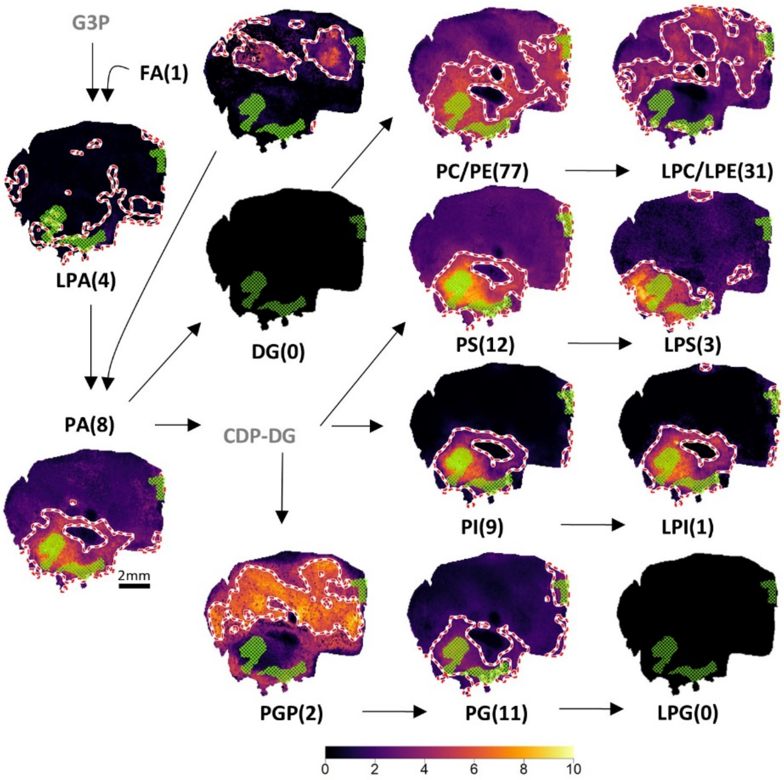

Supplementary Fig. 32. CPMs of lipid classes of the GB tissue section showcased in **Fig. 3ab**. CPMs enable spatial investigation of lipid synthesis and remodeling pathways in GBM tissue sections by visualizing structurally similar MOIs (e.g., lipid classes) within the same image space. Numbers in parenthesis refer to all detected and Metaspace-identified lipids at FDR ≤ 0.2 incl. alkali adducts. FA, fatty acid; PA, phosphatidic acid; DG, diglycerides; PC, phosphatidylcholine; LPC, lysophosphatidylcholine; LPE, lysophosphatidylethanolamine; PE, phosphatidylethanolamine; PI, phosphatidylinositol; CDP-DG, cytidine diphosphate diacylglycerol; LPG, lysophosphatidylglycerol; PG, phosphatidylglycerol; PGP, phosphatidylglycerolphosphate; LPS, lysophosphatidylserine; PS, phosphatidylserine.

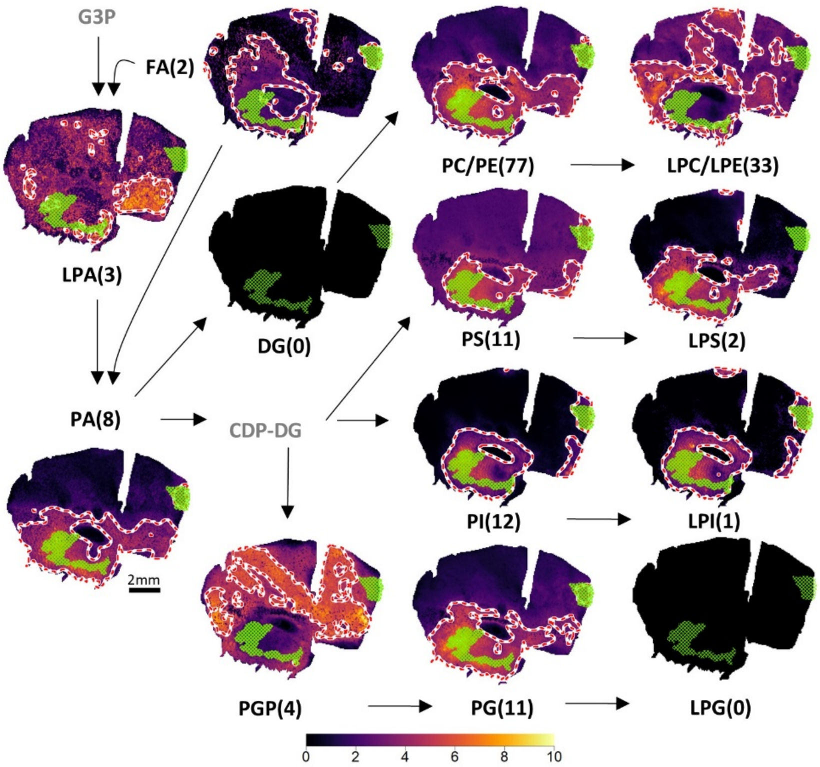

Supplementary Fig. 33. CPMs of lipid classes of a serial tissue section of the GB tissue section showcased in **Fig. 3ab**. CPMs enable spatial investigation of lipid synthesis and remodeling pathways in GBM tissue sections by visualizing structurally similar MOIs (e.g., lipid classes) within the same image space. Numbers in parenthesis refer to all detected and Metaspace-identified lipids at FDR ≤ 0.2 incl. alkali adducts. FA, fatty acid; PA, phosphatidic acid; DG, diglycerides; PC, phosphatidylcholine; LPC, lysophosphatidylcholine; LPE, lysophosphatidylethanolamine; PE, phosphatidylethanolamine; PI, phosphatidylinositol; CDP-DG, cytidine diphosphate diacylglycerol; LPG, lysophosphatidylglycerol; PG, phosphatidylglycerol; PGP, phosphatidylglycerolphosphate; LPS, lysophosphatidylserine; PS, phosphatidylserine.

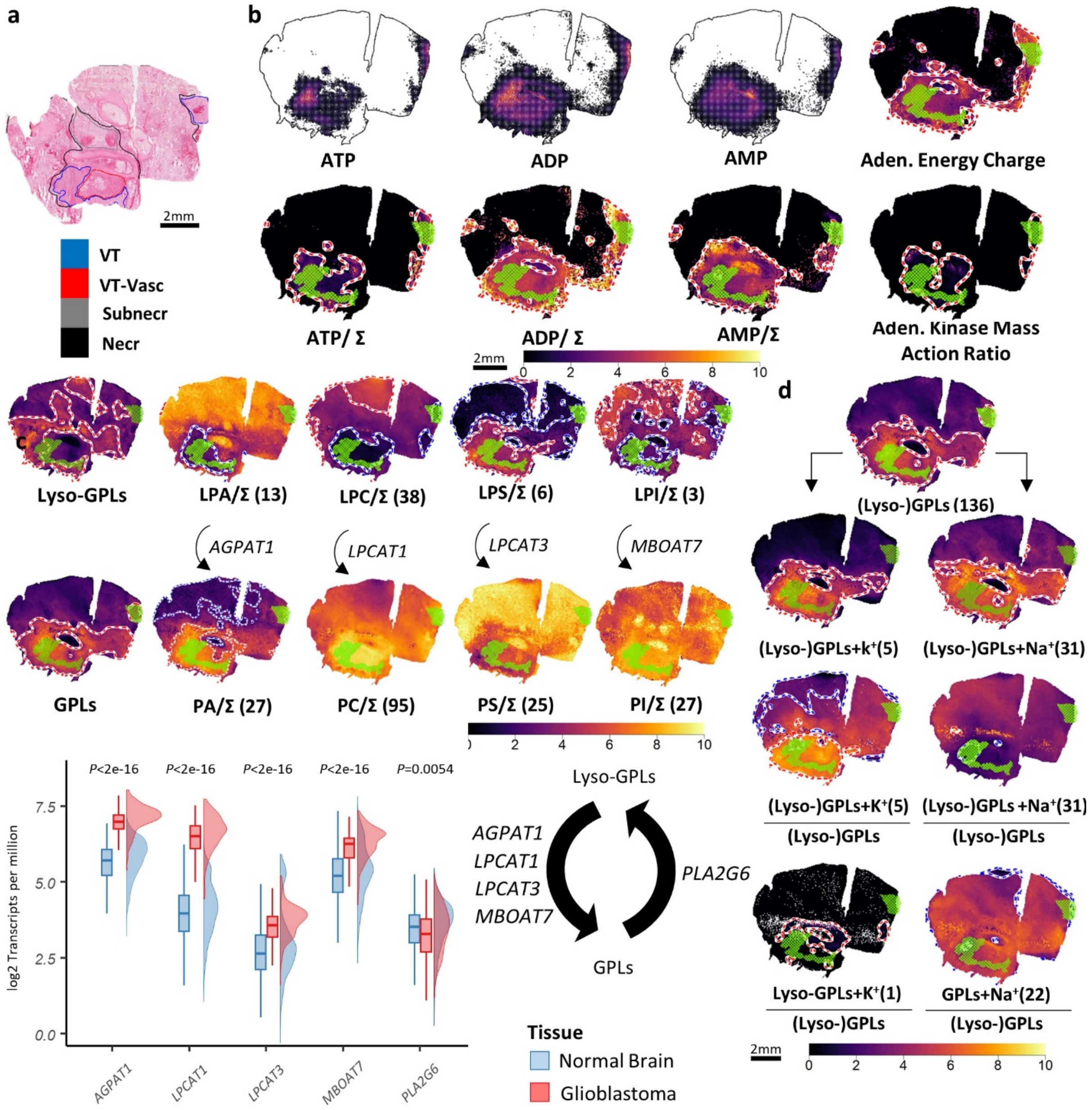

Supplementary Fig. 34. CPPMs for of an adjacent tissue section of GBM tissue section showcased in **Fig. 5**. **a)** H&E-stained image of the tissue section highlighting different GBM anatomical regions (VT: vital tumor; VT-Vasc: vital tumor with high vascularization; Subnecr: areas of pre-necrotic tissue; Necr: necrotic tissue). **b**) CPPMs enable basic arithmetic manipulations on SPPs of multiple MOIs. Green mesh indicates co-registered vital tumor regions (**a**). SPPs of $[ATP-H]^-$, $[ADP-H]^-$ and $[AMP-H]^-$ (≤0.2 FDR; upper row) and their sum-normalized CPPMs (bottom row; Σ = $[ATP-H]^-$ + $[ADP-H]^-$ + $[AMP-H]^-$). CPPMs also enable complex spatial quantitative scores such as adenylate energy charge (($[ATP-H]^-$+ 0.5 $[ADP-H]^-$)/($[ATP-H]^-$+$[ADP-H]^-$+$[AMP-H]^-$); top right) and adenylate kinase mass action ratio ($[ATP-H]^-$ $[AMP-H]^-$/$[ADP-H]^{-\,2}$; bottom right). **c**) CPPMs enable spatial investigation of GPL remodeling (Lands' cycle) in glioblastoma by collectively visualizing lipid classes. Upper panel: CPPMs of all lyso-GPLs and single classes (LPC, LPE, LPS, LPI; top row) compared to all GPLs and GPL classes (PC, PE, PS, PI; bottom row). Lyso- and non-lyso-GPL pairs are normalized to their sum (ex. for LPC and PC, Σ=LPCs plus PCs). Lower panel: the same data as shown in **Fig. 5c,** put here for convenience, please refer to the provided description in **Fig. 5c**. Boxplots indicate median (middle line), 25th, 75th percentile (box), and whiskers which extend to the most extreme data point which is no more than 1.5

times the length of the box away from the box. **d)** Analysis of the tissue's alkali ion milieu. Top: CPPMs for all (lyso-)GPLs, i.e. lyso-GPLs plus GPLs (FDR≤0.5). Left column from top: 1. CPPMs of (Lyso-)GPLs potassium adducts, 2. of (Lyso-)GPLs potassium adducts relative to the sum of all (lyso-)GPL adducts, and 3. CPPMs of only lyso-GPLs potassium adducts relative to the sum of all (Lyso-)GPL adducts. Right column: As left column but showing sodium adducts . PA, phosphatidic acid; PC, phosphatidylcholine; LPC, lysophosphatidylcholine; LPE, lysophosphatidyl-ethanolamine; PE, phosphatidylethanolamine; PI, phosphatidylinositol; LPG, lysophosphatidylglycerol; PG, phosphatidylglycerol; LPS, lysophosphatidylserine; PS, phosphatidylserine. Source data are provided as a Source Data file.

Supplementary Tables

Supplementary Table 1. Mass spectral data of the metabolites-of-interest (MOIs) shown in this study on the MALDI FTICR MSI data of the human glioblastoma (GB), mouse brain and porcine liver tissues.

MOI	Ionization	Molecular Formula	Calculated *m/z*	Observed *m/z*	Error (Da)	FWHM at MOI	R at MOI
SM(d36:4)	$[M+H]^+$	$C41H77N2O6P+H^+$	725.5592	725.5570	0.0022	0.0143	51k
PS(36:1)	$[M-H]^-$	$C42H80NO10P-H^-$	788.5447	788.5434	0.0013	0.0159	50k
SM(d34:2)	$[M+H]^+$	$C39H77N2O6P+H^+$	701.5592	701.5591	0.0001	0.0135	52k
ATP	$[M-H]^-$	$C10H16N5O13P3-H^-$	505.9884	505.9865	0.0019	0.0068	74k
ADP	$[M-H]^-$	$C10H15N5O10P2-H^-$	426.0221	426.0195	0.0026	0.005	85k
AMP	$[M-H]^-$	$C10H14N5O7P-H^-$	346.0558	346.055	0.0008	0.0035	100k
PE(20:1)	$[M+Na]^+$	$C25H48NO8P+Na^+$	544.3009	544.3007	0.0002	0.0087	62k
Heme	$[M+K]^+$	$C34H32FeN4O4+K^+$	655.1404	655.1408	0.0004	0.012	54k
PS(36:1)	$[M-H]^-$	$C42H80NO10P-H^-$	788.5447	788.5434	0.0013	0.0159	49k
SM(d36:1)	$[M+H]^+$	$C41H83N2O6P+H^+$	731.6061	731.6054	0.0007	0.0145	50k
PS(27:0)	$[M+H]^+$	$C33H64NO10P+H^+$	666.4340	666.4346	0.0006	0.0124	54k
Cer(d36:1)	$[M-H]^-$	$C36H71NO3-H^-$	564.5361	564.5337	0.0024	0.0084	68k
C24:1 sulfatide	$[M-H]^-$	$C48H91NO11S-H^-$	888.6240	888.6235	0.0005	0.0214	42k
PS(40:6)	$[M-H]^-$	$C46H78NO10P-H^-$	834.5290	834.5292	0.0002	0.0186	45k
(3'-sulfo) GalCer(38:1)	$[M-H]^-$	$C44H85NO11S-H^-$	834.5770	834.5779	0.0009	0.0186	45k
Tryptophan	$[M-H]^-$	$C11H12N2O2-H^-$	203.0815	203.0815	<0.0001	0.0013	161k
Imatinib	$[M+H]^+$	$C29H31N7O+H^+$	494.2662	494.2661	0.0001	0.003	165k

Supplementary References

1 Kanawati, B., Bader, T. M., Wanczek, K. P., Li, Y. & Schmitt-Kopplin, P. Fourier transform (FT)-artifacts and power-function resolution filter in Fourier transform mass spectrometry. *Rapid Commun Mass Spectrom* **31**, 1607-1615, doi:10.1002/rcm.7940 (2017).

2 Satopaa, V., Albrecht, J., Irwin, D. & Raghavan, B. Finding a "Kneedle" in a Haystack: Detecting Knee Points in System Behavior. *IEEE* doi:10.1109/ICDCSW.2011.20 (2011).

3 Enzlein, T. *et al.* Computational Analysis of Alzheimer Amyloid Plaque Composition in 2D- and Elastically Reconstructed 3D-MALDI MS Images. *Anal Chem* **92**, 14484-14493, doi:10.1021/acs.analchem.0c02585 (2020).

4 Friedrich, M. *et al.* Tryptophan metabolism drives dynamic immunosuppressive myeloid states in IDH-mutant gliomas. *Nature Cancer* **2**, 723–740, doi:https://doi.org/10.1038/s43018-021-00201-z (2021).

5 Abu Sammour, D. *et al.* Quantitative Mass Spectrometry Imaging Reveals Mutation Status-independent Lack of Imatinib in Liver Metastases of Gastrointestinal Stromal Tumors. *Sci Rep* **9**, 10698, doi:10.1038/s41598-019-47089-5 (2019).
