# Supplementary material for: Spatial probabilistic mapping of metabolite ensembles in mass spectrometry imaging
Source: Nat Commun. 2023 Apr 1;14:1823. doi: 10.1038/s41467-023-37394-z (PMC10067847; doi:10.1038/s41467-023-37394-z)
Supplement: Supplementary file 3 — Reporting Summary [file 41467_2023_37394_MOESM3_ESM.pdf]

nature portfolio

Corresponding author(s): Carsten Hopf

Last updated by author(s): Feb 20, 2023

Reporting Summary

Nature Portfolio wishes to improve the reproducibility of the work that we publish. This form provides structure for consistency and transparency in reporting. For further information on Nature Portfolio policies, see our Editorial Policies and the Editorial Policy Checklist.

Statistics

For all statistical analyses, confirm that the following items are present in the figure legend, table legend, main text, or Methods section.

n/a	Confirmed	
	x	The exact sample size (n) for each experimental group/condition, given as a discrete number and unit of measurement
	x	A statement on whether measurements were taken from distinct samples or whether the same sample was measured repeatedly
	x	The statistical test(s) used AND whether they are one- or two-sided<br>*Only common tests should be described solely by name; describe more complex techniques in the Methods section.*
x		A description of all covariates tested
	x	A description of any assumptions or corrections, such as tests of normality and adjustment for multiple comparisons
x		A full description of the statistical parameters including central tendency (e.g. means) or other basic estimates (e.g. regression coefficient) AND variation (e.g. standard deviation) or associated estimates of uncertainty (e.g. confidence intervals)
x		For null hypothesis testing, the test statistic (e.g. F, t, r) with confidence intervals, effect sizes, degrees of freedom and P value noted<br>*Give P values as exact values whenever suitable.*
x		For Bayesian analysis, information on the choice of priors and Markov chain Monte Carlo settings
	x	For hierarchical and complex designs, identification of the appropriate level for tests and full reporting of outcomes
x		Estimates of effect sizes (e.g. Cohen's d, Pearson's r), indicating how they were calculated

Our web collection on statistics for biologists contains articles on many of the points above.

Software and code

Policy information about availability of computer code

Data collection	All MALDI FTICR MSI data were acquired with solariX 7T XR (Bruker Daltonics) FTICR MRMS using Compass ftmsControl (Version 2.2) and flexImaging (Version 5.0) softwares (both Bruker Daltonics), MALDI timsTOF MSI data were acquired with timsToF fleX (Bruker Daltonics, Bremen, Germany) and all MALDI TOF MSI data were acquired with Rapiflex MALDI TOF MS (Bruker Daltonics) using FlexImaging 5.0 software (Bruker Daltonics). MSI data were converted into the open imzML format using SCiLS Lab Software version 2016a (Bruker Daltonics, Bremen, Germany).
Data analysis	Open source R packages MALDIquant (1.21), MALDIquantForeign (0.13), Matrix (1.2.18), spatstat (2.3.4), raster (3.5.15), ggplot2 (3.3.6) were used for analyzing MSI data in R (4.0.2). Open source R packages Bioconductor (3.14), ggplot2 (3.4.0), RColorBrewer (1.1-3), gridExtra (2.3), ggridges (0.5.4) were used for Transcript Expression Profiling of TCGA and GTEx Datasets in R (4.1). The Metaspace platform (metaspace2020.eu) was used for annotating MSI data. The methods described in this study are provided as an open source R package moleculaR (0.9.1) and the source code is available on Github via this link: https://github.com/CeMOS-Mannheim/moleculaR

For manuscripts utilizing custom algorithms or software that are central to the research but not yet described in published literature, software must be made available to editors and reviewers. We strongly encourage code deposition in a community repository (e.g. GitHub). See the Nature Portfolio guidelines for submitting code & software for further information.

Data

Policy information about availability of data

All manuscripts must include a data availability statement. This statement should provide the following information, where applicable:

- Accession codes, unique identifiers, or web links for publicly available datasets
- A description of any restrictions on data availability
- For clinical datasets or third party data, please ensure that the statement adheres to our policy

MALDI MSI Data of human GB tissue sections, mouse brain tissue sections and porcine tissue presented in this study is available on Metaspace (metaspace2020.eu) through the following link: https://metaspace2020.eu/project/abusammour-2022. The MALDI-TOF-MSI data of the APP NL-G-F Alzheimer's disease mouse model presented is available via ProteomeXchange with identifier PXD020824. The SwissLipids database is available through the Metaspace portal (metaspace2020.eu) and could also be downloaded as a separate file (swisslipids.org). Transcript Expression Profiles of TCGA and GTEx Datasets are available from Genomic Data Commons (https://gdc.cancer.gov) and Genotype-Tissue Expression dataset (https://gtexportal.org), respectively. Source data are provided with this paper.

Human research participants

Policy information about studies involving human research participants and Sex and Gender in Research.

Reporting on sex and gender	In the present study, tissue specimens from one patient were analyzed. We report sex of the patient in the methods scetion. Based on the type of analysis that we have performed, we anticipate that the results are applicable to both female and male patients equally.
Population characteristics	As reported above, tissue specimens from one patient were analyzed. Therefore covariant-relevant population characteristics do not apply.
Recruitment	Participants were recruited through the Heidelberg University Hospital and gave informed consent prior to study inclusion. Patients were selected based on thorough histological an genetic analyis including both IDH testing and Illumina EPIC mmethylation arry and only patients with a diagnosis of glioblastoma, IDH-wildtype based on the current 5th edition of the WHO classification were included in the study. This is to reduce bias trough misdiagnosis and clearly defines the analyzed study population based on recent guidelines.
Ethics oversight	The research is conducted in concordance with the declaration of Helsinki and was approved by the Ethics Committee at Heidelberg University, Germany (applications S-130/2022 and AFmu-207/2017)

Note that full information on the approval of the study protocol must also be provided in the manuscript.

Field-specific reporting

Please select the one below that is the best fit for your research. If you are not sure, read the appropriate sections before making your selection.

☒ Life sciences ☐ Behavioural & social sciences ☐ Ecological, evolutionary & environmental sciences

For a reference copy of the document with all sections, see nature.com/documents/nr-reporting-summary-flat.pdf

Life sciences study design

All studies must disclose on these points even when the disclosure is negative.

Sample size	The proposed computational methods were applied on single-tissue MALDI MSI data, each comprising ten to hundred thousands of spectral samples (MSI pixels, i.e. mass spectra per tissue slice). The number of "pixels" is a direct result of the chosen step-size (e.g. 20 µm) and the size of the tissue slice. Several examples have been provided in the manuscript, featuring different modalities, data types, and tissue samples. The spectral sample size for MALDI MSI data was as follows:<br>1. Two MALDI-FTICR-MSI data of human GB tissues each measured in positive and negative ion modes and each mode comprising ≈25k spectra (Fig. 3b, Fig. 5, Suppl. Fig. 27, Suppl. Fig. 29-34).<br>2. Six MALDI-FTICR-MSI data of mouse brain tissues each comprising ≈132k spectra (Fig. 3c, Suppl. Fig. 6, Suppl. Fig. 17, Suppl. Fig. 28).<br>3. Three sets of MALDI-FTICR-MSI data of IDH-mutant and -wildtype human GB tissues each set comprising ≈17k spectra (Suppl. Fig. 18).<br>4. MALDI-FTICR-quantification MSI data of porcine liver tissue comprising ≈3k spectra (Suppl. Fig. 16).<br>5. MALDI-TOF-quantification MSI data of gastrointestinal stromal tumor tissue comprising ≈5k spectra (Suppl. Fig. 20).<br>6. MALDI-timsTOF-MSI data of mouse brain tissue comprising ≈132k spectra (Suppl. Fig. 7, Suppl. Fig. 17).<br>7. MALDI-TOF-MSI data of mouse brain tissue comprising ≈132k spectra (Suppl. Fig. 8, Suppl. Fig. 17).<br>8. MALDI-TOF-MSI data of an APP NL-G-F Alzheimer's disease mouse model comprising ≈125k spectra (Fig. 3d, Suppl. Fig. 15).
Data exclusions	No data was excluded. For Suppl. Fig. 7 a MALDI-TOF dataset with sub-standard mass resolution was chosen on purpose. This is indicated in the corresponding Fig. legend as "sub-standard (used on purpose)".
Replication	The robustness of the proposed methods were tested against several MALDI MSI data featuring different modalities, data types, and tissue

	samples. All attempts at replication were successful. MALDI MSI experiments were replicated as follows:<br>1. MSI measurements and analysis of human GB tissues each measured in positive and negative ion modes were replicated once (Fig. 3b, Fig. 5, Suppl. Fig. 27, Suppl. Fig. 29-34).<br>2. MSI measurements and analysis of mouse brain tissues were replicated 8 times (Fig. 3c, Suppl. Fig. 6, Suppl. Fig. 17, Suppl. Fig. 28, Suppl. Fig. 7, Suppl. Fig. 17, Suppl. Fig. 8, Suppl. Fig. 17).<br>3. MSI measurements and analysis of IDH-mutant and -wildtype human GB tissues were done on three different tissue sets (Fig. 3e, Suppl. Fig. 18).<br>4. MSI measurement and analysis of porcine liver tissue was not replicated (Suppl. Fig. 16).<br>5. MSI measurement and analysis of gastrointestinal stromal tumor tissue was not replicated (Suppl. Fig. 20).<br>8. MSI measurement and analysis of an APP NL-G-F Alzheimer's disease mouse model was not replicated (Fig. 3d, Suppl. Fig. 15).
Randomization	This study does not provide statistical inference on cohorts of animals or human individuals. Randomization was therefore neither possible nor necessary. Internally, the poroposed MPM and CPPM methods use random permutations to generate complete spatial randomness models to be used as spatial null distributions of analytes' intensities. MPMs were also shown to be stable against 100 random permutations of the generated complete spatial randomness (CSR) models (Suppl. Fig. 25).
Blinding	MSI data of the glioblastoma sample (and technical replicate) was acquired prior to neuropathology annotation. MSI data, in turn, was unknown to the neuropathologist. Blinding considerations did not apply to all other datasets shown or used in this study as these were used to show the stability of results in inter-tissue and inter-slide setups and were not compared to orthogonal methods or judged by external operators.

Reporting for specific materials, systems and methods

We require information from authors about some types of materials, experimental systems and methods used in many studies. Here, indicate whether each material, system or method listed is relevant to your study. If you are not sure if a list item applies to your research, read the appropriate section before selecting a response.

Materials & experimental systems

n/a	Involved in the study
☒	☐ Antibodies
☒	☐ Eukaryotic cell lines
☒	☐ Palaeontology and archaeology
☐	☒ Animals and other organisms
☒	☐ Clinical data
☒	☐ Dual use research of concern

Methods

n/a	Involved in the study
☒	☐ ChIP-seq
☒	☐ Flow cytometry
☒	☐ MRI-based neuroimaging

Animals and other research organisms

Policy information about studies involving animals; ARRIVE guidelines recommended for reporting animal research, and Sex and Gender in Research

Laboratory animals	C57BL/6N mice.
Wild animals	The study did not include wild animals.
Reporting on sex	Sex-based analysis has not been performed since the study did not contain research/comparison of cohorts of animals.
Field-collected samples	The study did not include samples collected from the field.
Ethics oversight	For the study, we used leftover deep-frozen mice organs provided by the German Cancer Research Center, which were sacrificed to control breeding. Therefore ethical approval was not required.

Note that full information on the approval of the study protocol must also be provided in the manuscript.
